# Lmo4 synergizes with Fezf2 to promote direct *in vivo* reprogramming of upper layer cortical neurons and cortical glia towards deep-layer neuron identities

**DOI:** 10.1371/journal.pbio.3002237

**Published:** 2023-08-08

**Authors:** Torsten Felske, Chiara Tocco, Sophie Péron, Kawssar Harb, Christian Alfano, Chiara Galante, Benedikt Berninger, Michèle Studer

**Affiliations:** 1 Université Côte d’Azur, CNRS, Inserm, iBV, Nice, France; 2 Research Group “Adult Neurogenesis and Cellular Reprogramming”, Institute of Physiological Chemistry, University Medical Center Johannes Gutenberg University, Mainz, Germany; 3 Centre for Developmental Neurobiology, Institute of Psychiatry, Psychology & Neuroscience, King’s College London, London, United Kingdom; 4 MRC Centre for Neurodevelopmental Disorders, Institute of Psychiatry, Psychology & Neuroscience, King’s College London, London, United Kingdom; 5 Focus Program Translational Neuroscience, Johannes Gutenberg University, Mainz, Germany; ICM, FRANCE

## Abstract

*In vivo* direct neuronal reprogramming relies on the implementation of an exogenous transcriptional program allowing to achieve conversion of a particular neuronal or glial cell type towards a new identity. The transcription factor (TF) *Fezf2* is known for its role in neuronal subtype specification of deep-layer (DL) subcortical projection neurons. High ectopic *Fezf2* expression in mice can convert both upper-layer (UL) and striatal projection neurons into a corticofugal fate, even if at low efficiency. In this study, we show that *Fezf2* synergizes with the nuclear co-adaptor *Lmo4* to further enhance reprogramming of UL cortical pyramidal neurons into DL corticofugal neurons, at both embryonic and early postnatal stages. Reprogrammed neurons express DL molecular markers and project toward subcerebral targets, including thalamus, cerebral peduncle (CP), and spinal cord (SC). We also show that co-expression of *Fezf2* with the reprogramming factors *Neurog2* and *Bcl2* in early postnatal mouse glia promotes glia-to-neuron conversion with partial hallmarks of DL neurons and with *Lmo4* promoting further morphological complexity. These data support a novel role for *Lmo4* in synergizing with *Fezf2* during direct lineage conversion *in vivo*.

## Abstract 2

Direct neuronal reprogramming relies on the implementation of an exogenous transcriptional program to achieve conversion of a particular neuronal or glial cell type to a new identity. This study shows that the fate determinant transcription factor Fezf2 synergizes with the co-adaptor Lmo4 to reprogram upper layer cortical neurons or postnatal glia cells into corticofugal projection neurons.

## Introduction

While mechanisms driving the acquisition of specific neuronal class and subtype identities are increasingly investigated, the maintenance of such identity and conversely their degree of plasticity remains rather enigmatic [1]. Work over the last decade has challenged the view that neural cell identity is irrevocably fixed by demonstrating that fate-restricted neuronal progenitors and even early postmitotic neurons can be coaxed into neurons of distinct identities when appropriate transcriptional cues are provided [2–4], an experimental approach referred to as direct lineage reprogramming [5]. Likewise, different classes of glial cells, i.e., astrocytes, oligodendrocyte progenitor cells, and microglia can be converted into induced neurons by forced expression of neurogenic transcription factors (TFs) or regulatory *RNAs*, known to act as key regulators of cell fate during development [6,7]. However, it is still unclear to which extent induced neurons generated by direct lineage reprogramming acquire authentic molecular signatures of the desired neuronal subtype sharing similar developmental trajectories, function, and connectivity with subtype-specific projections.

Although direct lineage reprogramming still inspires cell replacement therapy, it also includes major limitations that need to be overcome. First, molecular and cellular features of the starter cell type may ease or impede the conversion process, and lineage-related cells might be easier to convert into each other as they share a common origin. For example, glial cells can be converted *in vitro* into functional neurons by overexpression of a single TF [8,9], whereas *in vivo*, single-factor reprogramming is much more limited, often requiring additional stimuli such as tissue injury and glial cell reactivation [10–13]. Moreover, the same reprogramming factor can trigger different outcomes when induced in different cell types. For example, Neurog2 has been shown to convert cortical astrocytes into glutamatergic-like cortical neuros [9,14], while thalamic and spinal astrocytes acquire thalamic relay neurons and spinal interneuron-like signatures, respectively [15,16], and fibroblasts even adopt a cholinergic motor neuron-like cell fate [17]. Moreover, successful reprogramming may require co-factors that negotiate critical transitions in the cellular metabolism [18,19]. Conversion of one postmitotic neuron subtype into another appears to be more complex and limited to the very early stages of postnatal life. For example, embryonic (E) 14.5 callosal projection neurons of cortical upper layers (ULs) II to IV could be converted into deep layers (DLs) V/VI subcortical projection neurons via the forced expression of the TF *Fezf2*, but failed at later stages, i.e., after postnatal (P) day 3 [2,4,20,21]. These observations point to the existence of a tight crosstalk between reprogramming factors and the cellular context in which they operate and suggest that the epigenetic signature of the starting cell population may limit cellular plasticity and their response to reprogramming factors [22]. Thus, current research aims at identifying reprogramming roadblocks whose removal could improve reprogramming efficiency and accuracy [7]. For example, during glia-to-neuron reprogramming, increased production of reactive oxygen species (ROS) can lead to cell death of induced neurons (iNs) per ferroptosis [18]. This has been partially overcome by allowing the expression of anti-cell death regulators, such as Bcl2, or by pharmacological treatments aimed at reducing ROS. Simultaneous expression of *Neurog2* and *Bcl2* induced the reprogramming of non-neuronal cells into immature DL pyramidal neurons [18].

To date, generating different neuronal subtypes of fully functional mature cells constitutes the major challenge in direct reprogramming [6,23]. TFs commonly used in direct neuronal reprogramming typically possess pioneering activity, such as transiently engaging closed chromatin to initiate transcriptional programs leading to cell fate changes [24,25]. However, they often fail to activate genes that are silenced by specific DNA and chromatin modifications [26]. Identifying co-factors that facilitate the binding of lineage-specific TFs on less accessible chromatin sites might improve reprogramming efficiency and specificity and stabilize neuronal identity. Therefore, one of the major aims of direct reprogramming becomes to identify novel factors conducive for generating specific neuronal subtypes.

Aiming at improving direct reprogramming efficiency and subtype specificity, first in immature neuronal cells and then in developing glial cells’ we used *Fezf2* as a well-established subcerebral determinant gene together with the co-adaptor *Lmo4*. Lmo4 is known to work as an epigenetic and subtype-specific factor by acting through an HDAC-dependent mechanism in de-repressing the *Ctip2* locus [27–31]. We show that *Lmo4* synergizes with *Fezf2* in converting UL neurons into DL subcortical projection neurons at a higher efficiency than previously reported [2,4]. We also find that *Fezf2* directs *Neurog2/Bcl2*-mediated reprogramming of early cortical glia into Ctip2-expressing iNs, while *Lmo4* further promotes their morphological complexity. Together, our data show that *Fezf2* synergizes with the co-adaptor *Lmo4* in lineage reprogramming in both neurons and glia towards a DL cortical neuron fate.

## Results

### *Fezf2* acts synergistically with *Lmo4* in converting upper- into deep-layer neurons during embryonic stages

Previous data showed that overexpression of *Fezf2* in striatal progenitors or young postmitotic cortical ULs II to IV neurons converts these neurons into DL (i.e., layer V/VI) corticofugal projection neurons (CFuPNs) [2,4,20,21,32]. Although reprogrammed neurons projected subcortically and acquired morphological and electrical properties characteristic of DL projection neurons, the efficiency of the cortical lineage conversion was between 20% and 30% dependent on the CFuPN marker used and strongly diminished postnatally [2,4]. We hypothesized that the nuclear co-adaptor and epigenetic factor *Lmo4* could be a good candidate for potentiating cell fate changes and improving conversion rates. Indeed, previous observations from our lab had shown that *Lmo4* overexpression leads to increased expression of the layer V marker Ctip2 in DL neurons [31].

**Fig 1:**
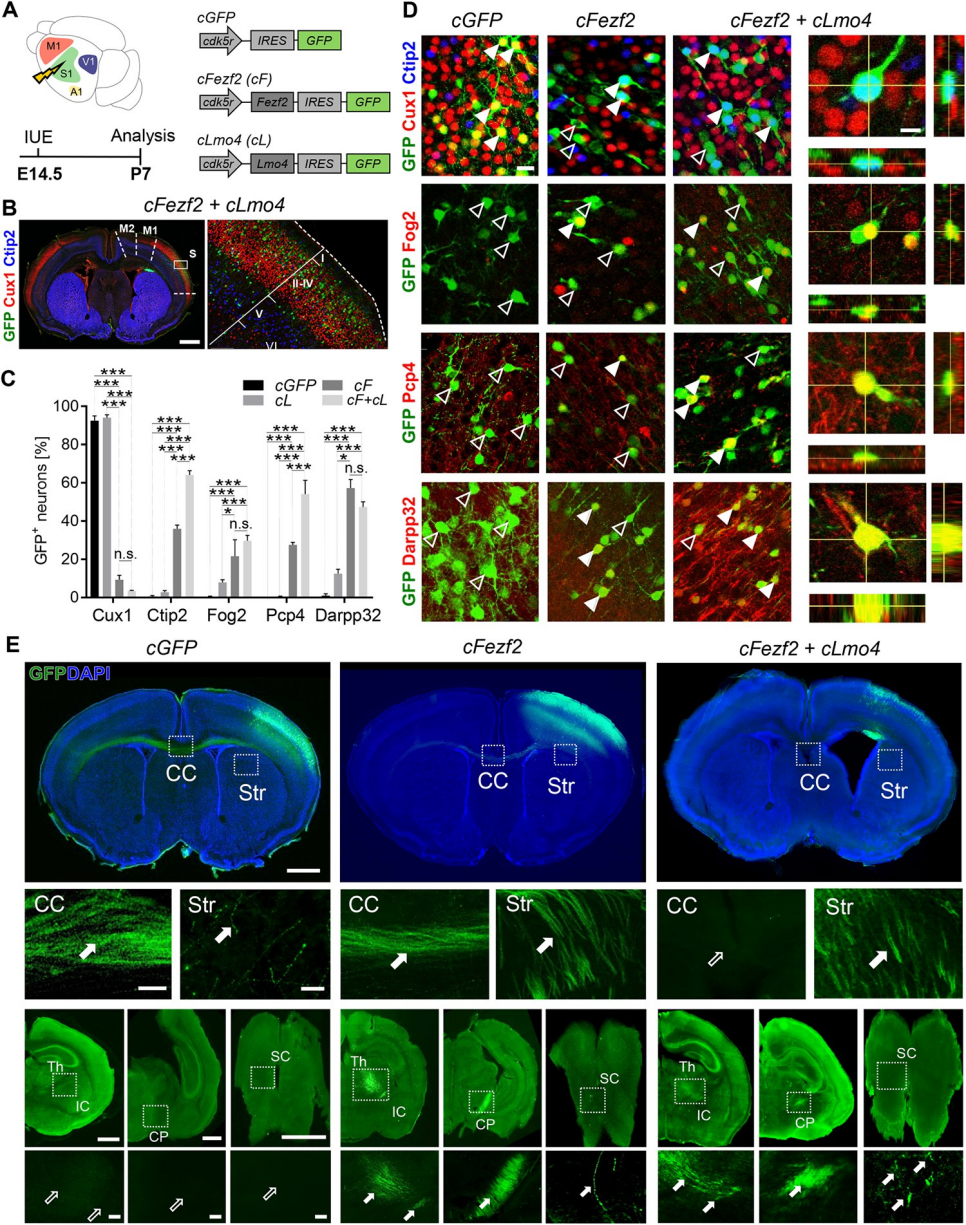
Synergistic effect of *Fezf2* and *Lmo4* in upper- to deep-layer neuron reprogramming. **(A)** Schematic representation of the experimental procedure and vectors. *cGFP*, *cLmo4 (cL)*, *cFezf2 (cF)*, or *cFezf2* and *cLmo4 (cF+cL)* plasmids were electroporated into E14.5 somatosensory (S1) embryonic cortices. Brains were collected at P7. **(B)** IF of GFP, UL marker Cux1, and DL V marker Ctip2 on a coronal slice of a *cFezf2* and *cLmo4*-electroporated brain. The white box indicates the magnification image on the right side. (**C**) Percentage of S1 electroporated UL neurons expressing upper vs. DL markers. (**D**) Representative images of Cux1, Ctip2, Fog2, Pcp4, and Darpp32 IF staining in electroporated brains. Full and empty arrowheads indicate whether GFP+ cells co-express or not, respectively, the marker. To the right, confocal images of high-magnification panels showing 3D reconstructions of double staining. Sidebars represent projections along the x–z axes (right) and the y–z axes (below). (**E**) Tract tracing of UL GFP+ axons upon electroporation of *cGFP, cFezf2* or *cFezf2* and *cLmo4* in P7 brains. In control cases, GFP+ axons cross the CC to reach the contralateral hemisphere. In *cFezf2*-electroporated brains, fewer GFP+ axons cross the CC, and many are found in the striatum (Str) and subcerebral targets, such as IC, thalamus (Th), CP, and SC. In *cFezf2* and *cLmo4*-electroporated brains, no projections are observed along the CC, and almost all GFP+ axons project through the Str to subcerebral targets. White boxes indicate the regions magnified in the panels below. Full and empty arrows indicate the presence or absence of axons, respectively. Scale bars: C = 1,000 μm (left, macro image) and 200 μm (right, magnified image); D = 20 μm; E = 1,000 μm (top row, macro images), 200 μm (magnified images). Results are expressed as mean ± SEM. Two-way ANOVA with Tukey’s post hoc correction was used for statistical analysis, *p < 0.05, **p < 0.01, ***p < 0.001. *n* = 3 brains for each plasmid. Extended data and statistics are listed in **S1 Data**. CC, corpus callosum; CP, cerebral peduncle; DL, deep layers; GFP, green fluorescent protein; IC, internal capsule; IF, immunofluorescence; UL, upper layers.

To test our hypothesis, we first performed *in utero* electroporation (IUE) to induce expression of our proposed reprogramming cocktail into embryonic wild-type (WT) mouse cortex. We took advantage of the *Cdk5r* gene promoter, which drives gene expression starting from the young postmitotic stage of neuronal maturation [33], to restrict ectopic expression of *Lmo4* and/or *Fezf2* to postmitotic neurons. The reporter gene *Green Fluorescent Protein* (*GFP*) was used as a readout allowing proper visualization of the electroporated cells. A *Cdk5r-GFP* (*cGFP*) plasmid was used as control, whereas the *Cdk5r-Fezf2-IRES-GFP (cFezf2)* and the *Cdk5r-Lmo4-IRES-GFP (cLmo4)* plasmids mediated *Fezf2* and *Lmo4* gain-of-function, respectively [2,31] (**Fig 1A**). Plasmids were then electroporated into the primary somatosensory (S1) cortex of embryonic age (E)14.5 WT embryos and electroporated brains were analyzed at postnatal stage P7 (**Figs 1** and **S1A**). *Cdk5r* promoter-driven gene expression became induced in migratory, postmitotic neurons and was then retained in differentiated and mature neurons (**Figs 1B** and **S1B**) [2,34]. A panel of upper- and deep-layer (hereinafter named UL and DL, respectively) molecular markers expressed at early or late cortical development was then assessed in electroporated brains: Cux1 for UL callosal neurons [35], Ctip2 and Pcp4 [36,37], Fog2 and Darpp32 for DL neurons [38–40]. To better define the precise laminar and cell-type specificity of the markers, we first performed double-immunostaining for Fog2, Pcp4, and Darpp32 with the well-described layer V marker Ctip2 [36] in S1 of WT non-electroporated brains (**S1C Fig**). We found that 74% of Ctip2+ high-expressing cells in layer V co-expressed Pcp4, while only 4% co-expressed Fog2 and 9% Darpp32. Conversely, Ctip2+ low-expressing cells, predominantly localized in layer VI, were strongly positive for Fog2 (70%), Darpp32 (80%), and to a minor extent for Pcp4 (34%) (**S1C Fig** and **S1 Data).** These data confirm that high expression of Ctip2 or Pcp4 is a reliable readout of layer V neuron identity, while elevated levels of Fog2 and Darpp32 support a layer VI identity.

Then, we evaluated the reprogramming potential of *Lmo4* alone before combining it with *Fezf2* (**S2A** and **S2B Fig**). Ectopic expression of c*Lmo4* alone in electroporated UL neurons revealed that the percentage of GFP+ neurons expressing Cux1 was very similar to c*GFP* controls at P7 (**Figs 1C, S2C** and **S2D and S1 Data**) and that *cLmo4* alone failed to induce any layer V markers in UL GFP+ neurons, even if a few GFP+ cells expressed the layer VI markers Fog2 and Darpp32 (**S2C** and **S2D Fig** and **S1 Data**). Accordingly, P7 tract-tracing analysis of GFP+ neurons in E14.5 *cLmo4*-electroporated brains revealed GFP+ axons projecting through the corpus callosum (CC) towards the contralateral hemisphere, with very few axons projecting ipsilaterally to the striatum, similarly to *cGFP*-electroporated brains (**S2E Fig**). No projections were observed towards subcerebral targets, such as the thalamus, cerebral peduncle (CP), and spinal cord (SC), indicating that ectopic expression of c*Lmo4* alone at E14.5 does not lead to any evident changes in molecular identity and axonal connectivity of UL neurons.

We next hypothesized that the combined activity of *Lmo4* (as a Ctip2 de-repressor) with *Fezf2* (as a Ctip2 inducer) would improve *Fezf2*-driven reprogramming efficiency of S1 UL neurons into CFuPNs. The *cFezf2* plasmid was electroporated together with *cLmo4* at E14.5 (**Fig 1A** and **1B**), and high Lmo4 and Fezf2 levels were confirmed in electroporated brains with almost 90% of GFP+ cells expressing both proteins (**S1A** and **S1B Fig**). Quantification of the percentage of GFP+ cells expressing UL and DL markers in *cFezf2-*electroporated brains showed an 83% decrease in Cux1-expressing cells compared to control c*GFP*-electroporated brains and a concomitant increase of Ctip2+ (35%), Fog2+ (21%), Pcp4+ (28%), and Darpp32+ (56%) neurons (**Fig 1C** and **1D** and **S1 Data**). Remarkably, the co-electroporation of *cFezf2* and *cLmo4* produced an almost double increase of GFP+ cells expressing Ctip2 (64%) and Pcp4 (54%), but no significant further changes of GFP+ cells expressing Cux1, Fog2, and Pcp4 cells when compared to *cFezf2-*electroporated cells (**Fig 1C** and **1D** and **S1 Data**), suggesting that Lmo4 endorses electroporated cells to predominantly acquire a layer V-like identity. Remarkably, in agreement with the changed molecular identities, GFP+ axons were observed in the striatum (Str), thalamus (Th), internal capsule (IC), CP, and to a minor extent even in the SC of both *cFezf2*- and double *cFezf2/cLmo4*-electroporated cortices (**Fig 1E** and **Table 1**). Interestingly, no callosal axons projecting contralaterally were found in *cFezf2/cLmo4*-electroporated brains, suggesting that induced co-expression of c*Lmo4* and c*Fezf2* in UL neurons strongly inhibited axon midline crossing. Taken together, these data indicate that *Lmo4* synergistically acts with *Fezf2* in reprogramming UL to DL neurons, particularly into layer V Ctip2+ Pcp4+ neurons, by inducing an almost complete redirection of UL projections to corticofugal targets.

### Reprogramming of upper into layer V neurons by *Fezf2* and *Lmo4* is more efficient in primary motor than somatosensory cortex

**Fig 2:**
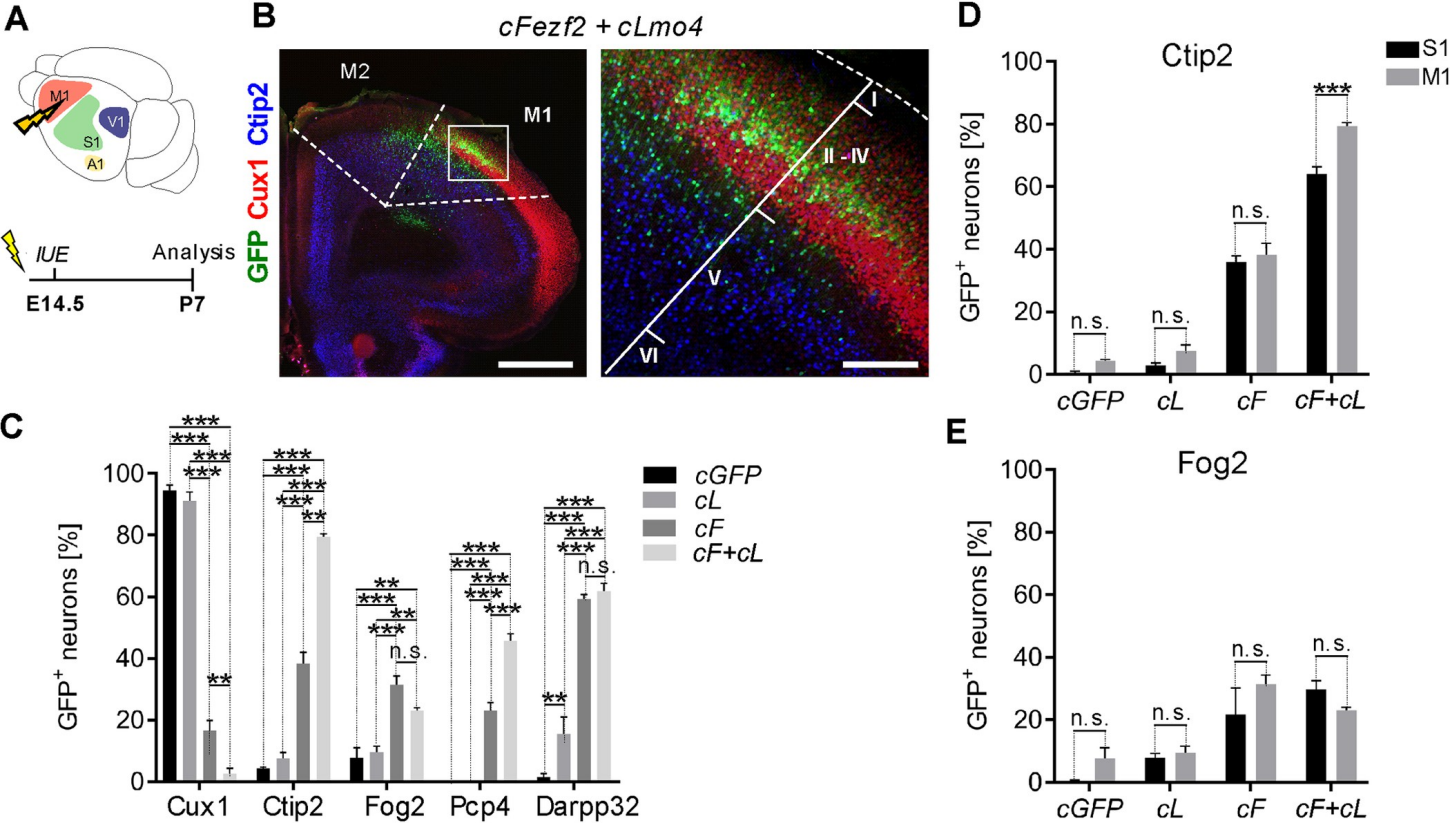
Reprogramming of ULs into layer V neurons by *Fezf2* and *Lmo4* is more efficient in motor than somatosensory cortex. (**A**) Schematic representation of the experimental procedure. *cGFP*, *cLmo4 (cL)*, *cFezf2 (cF)* or *cFezf2* and *cLmo4 (cF+cL)* plasmids were electroporated into E14.5 motor (M1) cortices. Brains were collected at P7. (**B**) IF of GFP, UL marker Cux1, and DL V marker Ctip2 on a coronal slice of a *cF+cL*-electroporated brain. The white box indicates the magnification image on the right side. (**C**) Percentage of M1 electroporated-UL neurons expressing UL vs. DL markers. (**D, E**) Percentage of S1 vs. M1-electroporated UL neurons expressing layer V marker Ctip2 (D) and layer VI marker Fog2 (E). Scale bars: B = 1,000 μm (left, macro image), 200 μm (right, magnified image). Results are represented as mean ± SEM. Two-way ANOVA with Tukey’s post hoc correction (2C) or two-way ANOVA with Sidak’s post hoc correction (2D-E) was used for statistical analysis. *p < 0.5, **p < 0.01, ***p < 0.001, ns = not significant. *n* = 3 brains for each plasmid. Extended data and statistics are listed in S2 Data. DL, deep layers; GFP, green fluorescent protein; IF, immunofluorescence; UL, upper layers.

We next investigated whether the cortical environment could impact UL to DL conversion. Since the primary motor area (M1) differs in its cytoarchitecture from S1 cortex by containing a higher number of layer V Ctip2+ neurons [41,42], we hypothesized that M1 might provide an even more conducive environment for layer V reprogramming of UL neurons. To examine this possibility, E14.5 embryos were electroporated with *cGFP*, *cLmo4*, *cFezf2*, or *cFezf2/cLmo4* plasmids into the presumptive motor area and brains analyzed at P7 (**Fig 2A**). Targeted GFP+ M1 postmitotic UL neurons (**Fig 2B**) were assessed for expression of UL and DL markers and then compared to S1-electroporated neurons (**Fig 2C–2E**). As for S1, ectopic expression of *cLmo4* alone did not result in any lineage conversion, but the combined expression of *cFezf2* with *cLmo4* in M1 resulted in a 92% reduction of the GFP/Cux1+ cells and an almost 80% increase of the GFP/Ctip2+ and 60% of the GFP/Pcp4+ cell populations, compared to the presence of 78% Cux1+ UL neurons versus 34% Ctip2+ and 23% Pcp4+ DL-like neurons in *cFezf2-*electroporated brains (**Fig 2C** and **S2 Data**). No statistical differences were observed in the number of layer VI Fog2+ and Darpp32+ cells between *cFezf2* and *cFezf2/cLmo4* GFP+ electroporated cells (**Fig 2C** and **S2 Data**), strongly supporting a synergistic effect of *Lmo4* with *Fezf2* in reconverting UL into layer V-like projection neurons. In addition, our data show that cell lineage conversion was more efficient in M1 than S1, particularly regarding the GFP/Ctip2+ cells that increased from 64% in S1 to almost 80% in M1 in double *cFezf2/cLmo4* UL GFP+ cells. No significant changes between S1 and M1 were observed for Fog2+ cells (**Fig 2D** and **2E** and **S2 Data**). These data strongly indicate that *Lmo4*/*Fezf2* co-expression leads to higher reprogramming efficiency in M1 than in S1.

This suggests that either the environment and/or the intrinsic cell competence of M1 are more conducive towards *Fezf2*-dependent layer V lineage conversion, in line with its expanded expression and a larger representation of subcerebral layer V projection neurons in M1 than in S1 during physiological development [43]. Even though M1 produced a slightly better conversion, S1 remained our choice of preference for the following experiments due to its high accessibility that grants more reliable and reproducible electroporation sites, and linked analyses.

### Reprogramming of UL into DL neurons by *Fezf2* and *Lmo4* is maintained until P35

**Fig 3:**
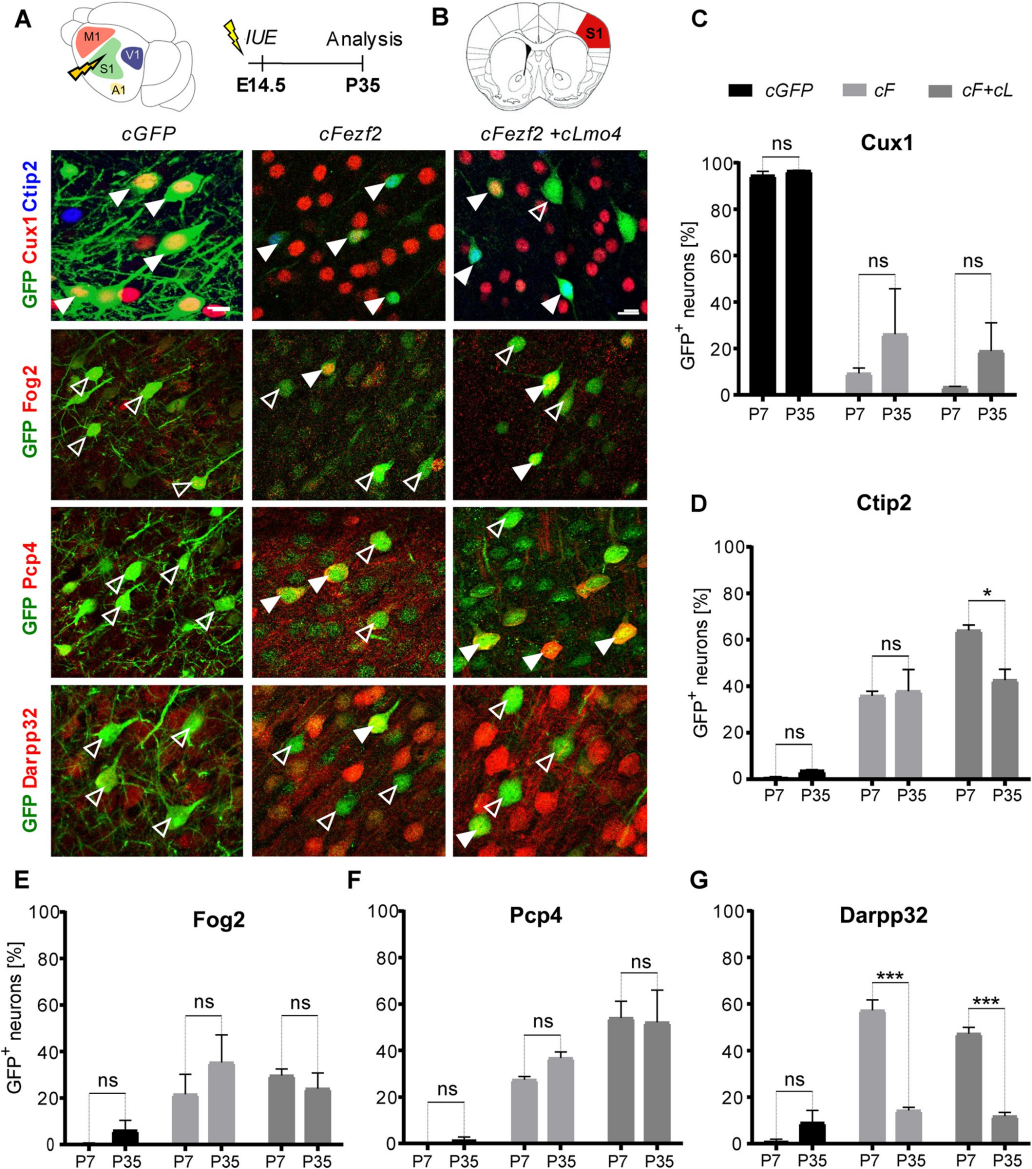
Reprogramming of UL into DL neurons by *Fezf2* and *Lmo4* is maintained until P35. **(A)** Schematic representation of the experimental procedure. *cGFP*, *cL*, *cF*, or *cF+cL* plasmids were electroporated into E14.5 somatosensory (S1) cortices. Brains were collected at P35. (**B**) Schematics of a coronal section of a brain showing in red the area (somatosensory cortex S1) from which the IF images below have been taken. Representative IF images of Cux1, Ctip2, Fog2, Pcp4, and Darpp32 staining in *cGFP*, *cF*, and *cF+cL*-electroporated brains. Full and empty arrowheads respectively indicate whether GFP+ cells co-express the indicated marker. (**C–G**) Percentage of S1-electroporated UL neurons expressing UL vs. DL markers compared between P7 and P35 brains. Scale bars: B = 20 μm. Results are represented as mean ± SEM. Two-way ANOVA with Sidak’s post hoc correction was used for statistical analysis. *p < 0.5, ***p < 0.001, ns = not significant. *n* = 3 brains for each plasmid. Extended data and statistics are listed in **S3 Data**. DL, deep layers; IF, immunofluorescence; UL, upper layers.

To assess whether the neuronal lineage conversion was a transient effect or stably maintained at later stages, E14.5 embryos were electroporated with *cGFP*, *cLmo4*, *cFezf2*, or *cFezf2/cLmo4* plasmids (**Fig 3A**) and the percentage of GFP+ neurons expressing UL and DL markers was determined at P35 and compared with those obtained at P7 (**Fig 3B–3G**). As expected, in control *cGFP-*electroporated brains, Cux1 expression was detected in almost all GFP+ UL neurons, both postnatally (P7) and at more mature stages (P35). In contrast, the number of GFP/Cux1+ cells among *cFezf2-* and *cFezf2/cLmo4-*electroporated UL neurons remained consistently low at P35 (**Fig 3C** and **S3 Data**). Similarly, no changes in the number of GFP/Fog2+ and GFP/Pcp4+ cells were observed between P7 and P35 in control, *cFezf2*, and *cFezf2/cLmo4-*electroporated brains (**Fig 3E** and **3F** and **S3 Data**). Conversely, the number of GFP/Darpp32-expressing significantly dropped in *cFezf2* and *cFezf2/cLmo4-*electroporated brains (**Fig 3G** and **S3 Data**). Finally, the rate of GFP/Ctip2+ UL neurons remained constant between P7 and P35 in both control *cGFP*- and *cFezf2*-electroporated brains (between 36% and 38%), whereas it slightly decreased from 64% to 43% in *cFezf2/cLmo4-*electroporated UL neurons (**Fig 3D** and **S3 Data**). Overall, our data show that most of the E14.5-reprogrammed UL neurons keep their newly acquired layer V/VI-like molecular signature over time.

### Postnatal induction of *Fezf2* and *Lmo4* expression

**Fig 4:**
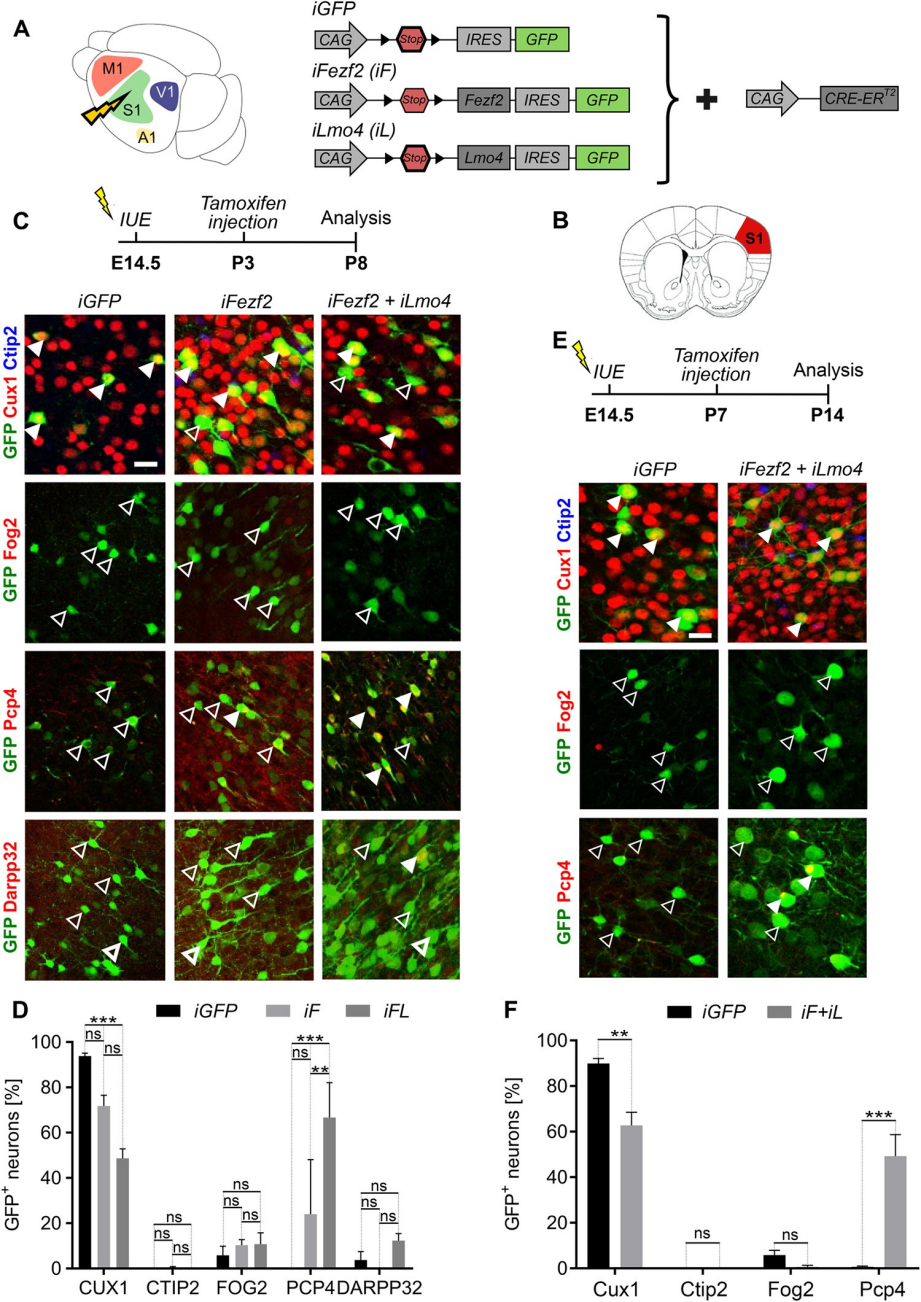
Postnatal induction of *Fezf2* and *Fezf2/Lmo4* expression promotes layer V and inhibits UL neuron identity. (**A**) Representation of the experimental vectors used in B and D. *iGFP*, *iFezf2 (iF)* or *iFezf2*, and *iLmo4 (iF+iL)* were electroporated into E14.5 somatosensory (S1) cortices. (**B**) Schematics of a coronal section of a brain showing in red the area (somatosensory cortex S1) from which the IF images in C and E have been taken. (**C**) Gene expression was induced at P3 by tamoxifen subcutaneous injection. Brains were collected at P8. Representative IF images of Cux1, Ctip2, Fog2, Pcp4, and DarppP32 staining in *iGFP*, *iF*, and *iF+iL*-electroporated brains. Full and empty arrowheads respectively indicate GFP+ cells co-expressing or not the indicated marker, respectively. (**D**) Percentage of S1 electroporated UL neurons expressing UL vs. DL markers. (**E**) Gene expression was induced at P7 by tamoxifen subcutaneous injection. Brains were collected at P14. *iGFP*, *iFezf2 (iF)* or *iFezf2* and *iLmo4 (iF+iL)* together with *pCAG-CRE-ER^T2^* were electroporated into the E14.5 S1 cortex. Representative IF images of indicated marker staining in *iGFP-* and *iFL*-electroporated brains. Full and empty arrowheads indicate GFP+ cells co-expressing or not the indicated marker. (**F**) Percentage of S1-electroporated UL neurons expressing UL vs. DL markers. Scale bars: B, E = 20 μm. Results are represented as mean ± SEM. Two-way ANOVA with Sidak’s post hoc correction was used for statistical analysis.**p < 0.01, ***p < 0.001, ns = not significant. *n* = 3 brains for each plasmid. Extended data and statistics are listed in **S4 Data**. DL, deep layers; GFP, green fluorescent protein; IF, immunofluorescence; UL, upper layers.

Given the high efficiency of *Fezf2* and *Lmo4* in reprogramming postmitotic UL neurons at embryonic stages, we next tested their potential at postnatal stages (**Fig 4**). To this purpose, we subcloned *Fezf2* or *Lmo4* into the inducible vector *pCAG-fl-mutCherry-fl-IRES-EGFP* [44] resulting in *pCAG-Ind-GFP (iGFP)*, *pCAG-Ind-Fezf2 (iFezf2)*, and *pCAG-Ind-Lmo4 (iLmo4)* (**Fig 4A**). All vectors were co-electroporated with the *pCAG-CRE-ER*^*T2*^ plasmid at E14.5, and 4-hydroxytamoxifen (TAM) was administrated at different postnatal stages to drive *Cre*-recombinase activation [45].

To validate our system and confirm that *iFezf2* and *iLmo4* could drive the expression of their respective proteins in electroporated GFP+ cells, TAM was injected at P3 in E14.5-electroporated mice and brains were collected at P8 (**S3A** and **S3B Fig**). Almost all cells were expressing Fezf2 and Lmo4 in TAM-induced neurons (**S3C Fig)**. To ensure that these vectors were not inducing their respective proteins without TAM administration, we co-electroporated an mCherry, *Cre*-independent plasmid together with *iFezf2/iLmo4* and *pCAG-CRE-ER*^*T2*^ and analyzed the expression of GFP (Cre-dependent) and mCherry (Cre-independent) reporters. No GFP signal was found in mCherry^+^ cells (**S3D Fig**), demonstrating that no unspecific activation of the inducible plasmids had occurred.

Next, we electroporated embryo brains at E14.5 with either *iGFP*, *iFezf2*, or *iFezf2/iLmo4* and induced expression of these genes at P3 via injection of TAM (**Fig 4A–4C**). Even at that postnatal stage, *iFezf2/iLmo4* expression resulted in a 45% decrease of GFP+ cells expressing Cux1 as compared to control *iGFP* and in a 23% reduction compared to *iFezf2* induction in S1 cortex (**Fig 4B–4D** and **S4 Data**). In line with previous observations [2,4], postnatal induction of either *iFezf2* or *iFezf2/iLmo4* did not lead to a significant increase in the Ctip2+ cell population, nor did it influence the number of Fog2+ and Darpp32+ cells in all electroporated conditions (**Fig 4C** and **4D** and **S4 Data**). Instead, expression of *iFezf2/iLmo4* was able to boost the number of GFP+ cells expressing the late layer V marker Pcp4 to 67% when compared to *iFezf2* alone, which only induced 24% of GFP+ cells to express Pcp4 (**Fig 4C** and **4D** and **S4 Data**). This implies that the co-expression of *Lmo4* allowed substantially more GFP+ cells to express Pcp4. To further assess the reprogramming potential of *iFezf2*/*iLmo4* at later stages, we repeated the same experiment by injecting TAM at P7 and analyzing the brains at P14 (**Figs** **4E** and **4F** and **S4**) and TAM at P10 and analyzing the brains at P21 (**S5 Fig**). Interestingly, we could observe a reduction of GFP/Cux1+ cells to 63% and a remarkable increase of GFP/Pcp4+ to 49% in *iFezf2/iLmo4*-induced cells compared to control *iGFP* upon injection of TAM at P7 (**Fig 4E** and **F** and **S4 Data**).

These data show that the synergistic effect of *iFezf2* and *iLmo4* allows a higher number of cells to adopt a DL-like identity when compared to *iFezf2* alone (**Fig 4**) [2,4]. Notably, the percentage of reprogrammed GFP+ cells expressing Pcp4 was even higher than in embryonic reprogramming experiments, confirming a preferentially postnatal expression pattern for Pcp4 in CFuPN development, as previously suggested [36]. Taken together, the reprogramming cocktail of *iFezf2* and *iLmo4* revealed remarkable molecular identity alterations when induced at P3 and P7 in UL neurons, confirming a robust synergistic effect of both factors in reprogramming postnatal cortical projection neurons.

### Postnatal induction of double *iFezf2/iLmo4* expression drives upper-layer neurons to change their axonal projections toward subcerebral targets

**Fig 5:**
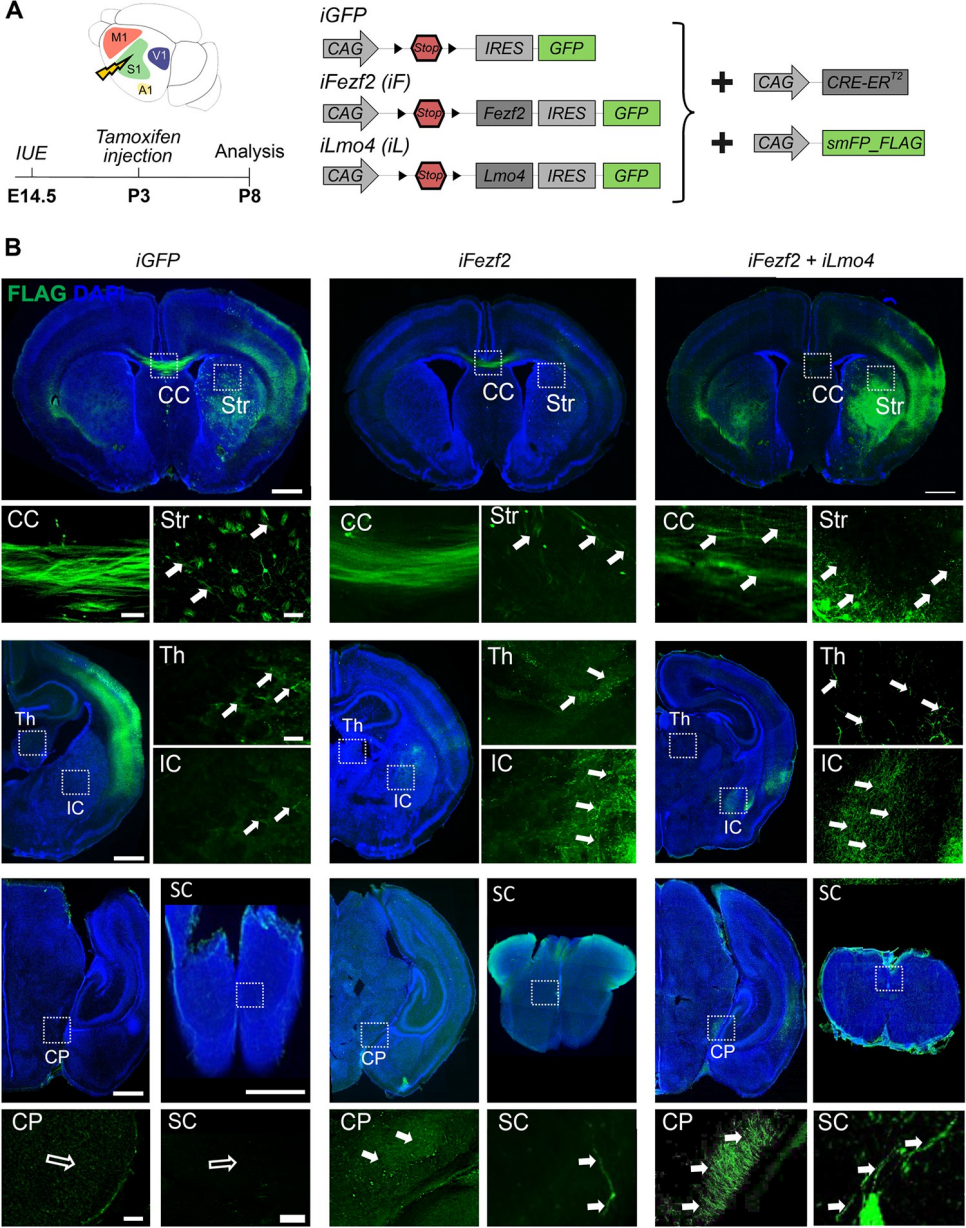
Postnatal induction of *Fezf2* and *Lmo4* expression drives UL neurons to change their axonal projections toward subcerebral targets. (**A**) Schematic representation of the experimental procedure and vectors. *iGFP*, *iFezf2 (iF)* or *iFezf2*, and *iLmo4 (iF+iL)* together with *pCAG-CRE-ER^T2^* were electroporated into E14.5 somatosensory (S1) cortices. The *smFP-Flag* reporter plasmid was co-electroporated to facilitate axonal tracing. Gene expression was induced at P3 by tamoxifen subcutaneous injection. Brains were collected at P8. (**B**) Tract tracing of UL FLAG+ axons upon electroporation of indicated plasmids. FLAG+ axons were found crossing the CC and reaching the striatum (Str), thalamus (Th), and IC in all conditions but projecting towards the CP and SC only in *iF-* and *iF+iL*-electroporated brains. Note that GFP+ cells in the Str of *iGFP* and *iFL* are electroporated ectopic cells and not axons. White boxes indicate regions magnified in the panels below or aside. Full and empty arrows indicate the presence or absence of axons, respectively. Scale bars: B = 20 μm. *n* = 3 brains for each plasmid. CC, corpus callosum; CP, cerebral peduncle; GFP, green fluorescent protein; IC, internal capsule; SC, spinal cord; UL, upper layers.

To further assess the potential of *Fezf2* and *Lmo4* in converting UL to DL neurons at postnatal stages, we followed reprogrammed GFP+ axonal projections. To facilitate tract tracing and obtain a stronger axonal signal of electroporated neurons in postnatal stages, we took advantage of the *pCAG-smFP_FLAG* plasmid, which encodes multiple copies of the epitope tag FLAG [46]. The so-called “spaghetti monster” protein does not emit any fluorescence on its own but can be easily detected by using an anti-FLAG antibody. After co-electroporating either *iGFP*, *iFezf2*, or *iFezf2/iLmo4* together with the *pCAG-CRE-ER*^*T2*^ and *pCAG-smFP_FLAG* plasmids at E14.5, transgene expression was induced by TAM injection at P3, P7, and P10, and brains collected at P8, P14, and P21, respectively (**Figs 5A** and **S4** and **S5**). In all conditions, we could detect FLAG+ axons along the CC and in the striatum, although fibers along the CC of *iFezf2/iLmo4*-electroporated brains appeared less organized (**Fig 5B**, see arrows in CC). Remarkably, several axons were detected in the IC, CP, and SC of *iFezf2-* and *iFezf2/iLmo4*-electroporated brains with FLAG+ bundles more prominent in the CP and SC upon *iFezf2/iLmo4* than just *iFezf2* induction (**Figs 5B** and **S4** full arrows in CP and SC), suggesting a higher efficiency of axonal rerouting in the presence of *Lmo4* (**Table 1**). Few FLAG+ axons were found in the thalamus in all conditions (**Figs 5** and **S4** and **S5**), indicating that reprogrammed neurons tend to follow a subcerebral trajectory rather than a corticothalamic one, in line with the higher number of GFP/Pcp4+ layer V-like neurons as compared to the number of GFP/Fog2+ and GFP/Darpp32+ layer VI-like neurons (**Fig 4**). Compared to P3, TAM induction at P7 and P10 still promoted some FLAG+ axons to reach the CP, and particularly upon *iFezf2/iLmo4* expression to reach the SC, although at progressively lower efficiency (**Table 1** and **S4** and **S5 Figs**). Finally, we challenged the competence of UL neurons to undergo reprogramming at even later postnatal stages by inducing expression of *iGFP*, *iFezf2*, or *iFezf2/iLmo4* at P21 and collecting the electroporated brains at P35 (**S6A Fig**). Since no DL markers were induced at this stage, we limited our analysis to FLAG+ axonal trajectories. Induction at P21 showed axonal projections crossing the CC with ipsilateral projections reaching the striatum in all conditions (**S6B Fig**). While no signal was detected in the thalamus, FLAG+ axons could be observed in the IC of all electroporated conditions. Notably, a clear axonal signal was detected in the CP and SC of *iFezf2-* and even stronger in that of *iFezf2/iLmo4-*induced brains (**Table 1**), indicating that even at this late postnatal stage reprogrammed UL neurons can still be instructed to change their axonal projections towards subcerebral targets.

### *Fezf2* promotes acquisition of deep-layer-like hallmarks in neurons induced from glia

**Fig 6:**
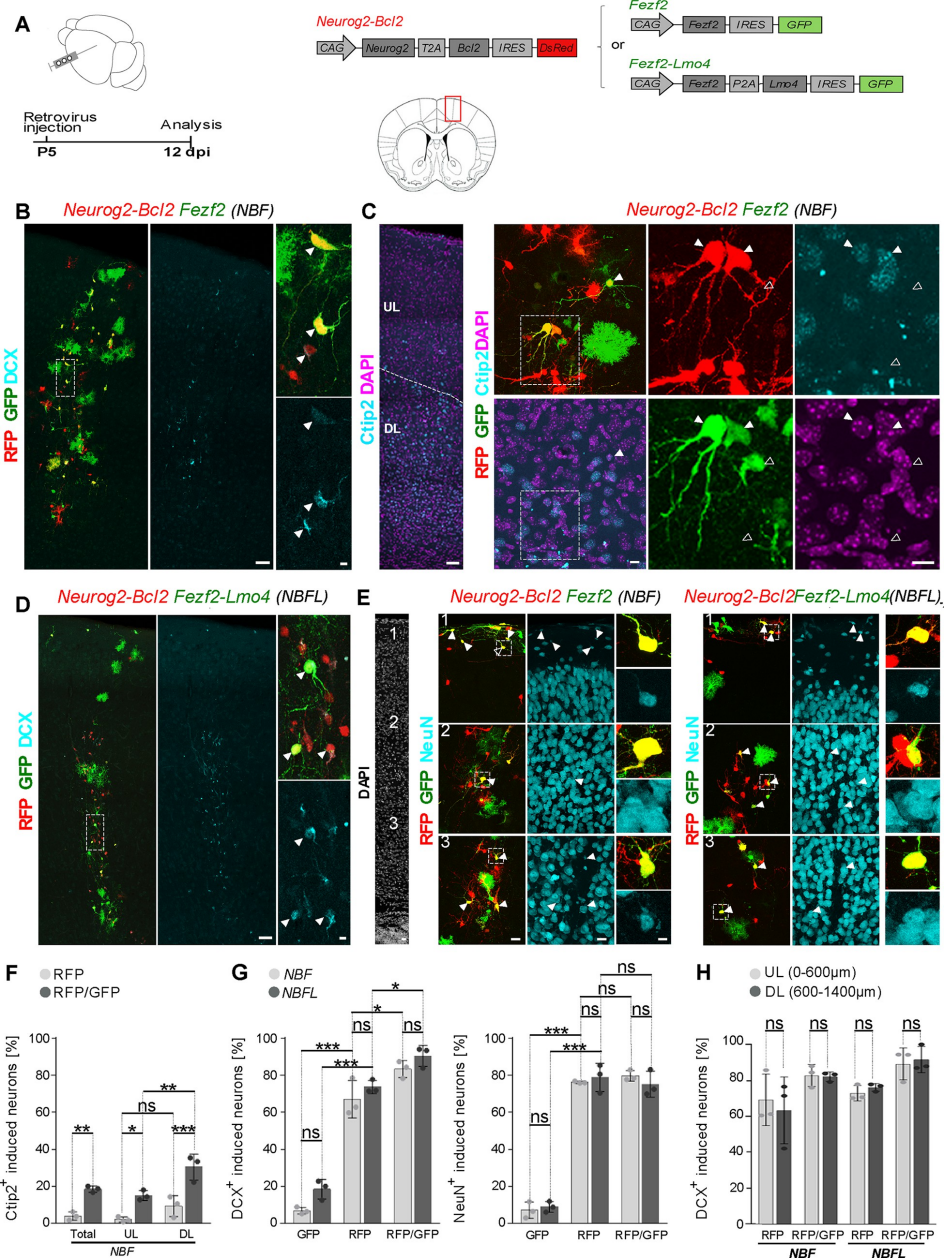
*Fezf2* promotes a layer V-like fate in neurons induced from glia by *Neurog2-Bcl2*. (**A**) Schematic representation of the experimental design. Postnatal day 5 (P5) proliferative glia was targeted for reprogramming by injection of retroviruses encoding *Neurog2-Bcl2-DsRed (NB)* together with *Fezf2-GFP (NBF)* or *Fezf2-Lmo4-GFP (NBFL)*. Analysis was performed 12 days later. Middle, schematics of a coronal section of a brain outlined in red the area of imaging. (**B**) Numerous *NB*-and *NBF*-transduced cells express the immature neuronal marker DCX. Arrowheads show examples of DCX-expressing reprogrammed neurons. (**C**) Deep cortical (DL) layers are enriched in Ctip2-positive cell nuclei as compared with ULs. Some *NB*- and *NBF*-transduced cells express Ctip2. Solid-line arrowheads and dotted-line arrowheads show examples of Ctip2-positive and Ctip2-negative transduced cells, respectively. (**D**) Numerous *NB*- and *NBFL*-transduced cells express the immature neuronal marker DCX. Arrowheads show examples of DCX-expressing reprogrammed neurons. (**E**) In all cortical layers, numerous *NB- NBF-* and *NBFL*-transduced cells express the mature neuronal marker NeuN. Arrowheads show examples of NeuN-expressing reprogrammed neurons. (**F**) Quantification of the percentage of Ctip2 expressing cells among *NB*- and *NBF*-transduced cells. The percentage of Ctip2-expressing cells among *NBF*-transduced cells is increased as compared with *NB*-transduced cells, predominantly in deep cortical layers. Mean ± SD; Welch-ANOVA test; *n* = 3 brains, 676 cells analyzed. (**G**) Quantification of the percentage of DCX-expressing (left) or NeuN-expressing (right) cells among GFP+ (*F* or *FL*), RFP+ (*NB*), and RFP+GFP+ (*NBF* or *NBFL*) cells. The percentage of DCX-expressing reprogrammed neurons is moderately increased in *NBF*- and *NBFL*-transduced cells as compared with NB-cells. Mean ± SD; one-way ANOVA followed by Tukey post hoc test; *n* = 3 brains/retrovirus combination, 2,827 (DCX), and 1,550 (NeuN) cells analyzed. (**H**) Quantification of the percentage of RFP+ (*NB*) and GFP+RFP+ (*NBF* or *NBFL*) cells expressing DCX at different depth relative to the cortical surface indicates similar reprogramming efficiency in upper and deep cortical layer. Mean ± SD; Kruskal–Wallis test; *n* = 3 brains/retrovirus combination, 2,324 cells analyzed. Extended data and statistics are listed in **S5 Data**. DL, deep layers; GFP, green fluorescent protein; UL, upper layers.

We next investigated whether the subtype specifying roles of *Fezf2* and *Fezf2/Lmo4* could extend beyond the cellular context of neuron-to-neuron conversion and work also in glia-to-neuron reprogramming. Towards this end, we injected retroviruses encoding *Fezf2* or *Fezf2/Lmo4* (*CAG-Fezf2-IRES-GFP or CAG-Fezf2-P2A-Lmo4-IRES-GFP*) in combination with a retrovirus encoding *Neurogenin2* (*Neurog2*) and *B-cell lymphoma 2* (*Bcl2*) (*CAG-Neurog2-T2A-Bcl2-IRES-DsRed)*, previously shown to reprogram cortical glia into induced neurons [15], into the cerebral cortex of P5 mice. Moloney murine leukemia virus (MMLV)-based retroviruses were used for gene delivery in the postnatal cerebral cortex with the rationale that they selectively transduce cells undergoing mitosis [47] and thereby enable specific targeting of cortical glia undergoing proliferation [48]. Indeed, we have recently shown that retrovirus-targeted cells comprise by and large astrocytes (approximately 65%) and oligodendrocyte progenitors (roughly 35%), with very few other targeted cells [49] (**Fig 6**). First, we assessed overall glia-to-neuron conversion following transduction with *Neurog2-Bcl2* and/or *Fezf2* (*NBF*) 12 days after injection (**Fig 6A**). Consistent with the previously reported reprogramming of immature glia and adult reactive glia by *Neurog2-Bcl2* (*NB*) [15,18] into induced neurons (iNs), we found *NB*-transduced cells (RFP+ cells) expressing doublecortin (DCX) (**Fig 6B** and **6G**) indicating that retrovirus-induced conversion involved an immature neuron-like stage. In contrast, *Fezf2* expression alone (GFP+) had only a marginal effect on the number of DCX+ cells among the retrovirus-targeted cells and most cells exhibited a glial morphology (**Fig 6B** and **6G**). However, the percentage of cells losing glial morphology and expressing DCX increased to more than 80% following co-expression of *Fezf2* together with *Neurog2* and *Bcl2* (*NBF*) (RFP+/GFP+ cells in **Fig 6B** and **6G** and **S5 Data**). For both *NB*- and *NBF*-transduced cells, the conversion rate (i.e., DCX+ among reporter+ cells) was not affected by their depth relative to the cortical surface (**Figs 6H** and **S7** and **S5 Data**), suggesting that residing in a particular cortical layer does not affect reprogramming efficiency. We next assessed whether iNs acquired a DL-like identity by assessing Ctip2 expression (**Fig 6C** and **6F**). As expected, endogenous Ctip2 immunoreactivity was predominantly detected in nuclei located in deep cortical layers (**Fig 6C**). Our analysis revealed that less than 5% of the *NB*-transduced cells expressed Ctip2 (**Fig 6C** and **6F** and **S5 Data**). Strikingly, the proportion of cells expressing Ctip2 was more than 4 times higher among *NBF*-transduced cells as compared with *NB*-transduced cells (**Fig 6C** and **6F** and **S5 Data**). Intriguingly, *Fezf2*-induced specification into Ctip2+ iNs seemed to be facilitated in deep cortical layers enriched in endogenous Ctip2+ neurons. In fact, 30% of *NBF*-transduced cells located in deep cortical layers were Ctip2+ as compared to 15% of *NBF*-transduced cells in upper cortical layers (**Fig 6F** and **S5 Data**). We next analyzed reprogramming following transduction with *NB* together with *Fezf2-Lmo4* (*NBFL*). Co-expression of *Fezf2* and *Lmo4* in the absence of *Neurog2* and *Bcl2* was highly inefficient in inducing DCX expression in transduced cells (**Fig 6D** and **6G**). However, as seen with *NBF*, we found that upon *NBFL* expression reprogramming into DCX+ iNs was very efficient and indistinguishable between upper and deep cortical layers (**Fig 6H**). The percentage of DCX+ cells was increased to 83% among *NBFL*-transduced cells as compared to *NB*-transduced cells, while it was very similar to *NBF* overexpression (**Fig 6D** and **6G** and **S5 Data**). Our analysis further showed that many iNs expressed the mature neuronal marker NeuN throughout all cortical layers, without significant differences between *NB*, *NBF*, or *NBFL* conditions, while *Fezf2* or *Fezf2/Lmo4* alone failed to induce conversion into NeuN+ cells (**Fig 6E** and **6G**). Together, our data indicate that while *NB* can reprogram P5 proliferative cortical glia with good efficiency, the additional expression of *Fezf2* (alone or in combination with *Lmo4*) further enhances glia-to-neuron conversion and, importantly, instructs iNs to acquire layer V-like molecular hallmarks.

### Increased dendritic complexity of iNs co-expressing *Fezf2* and *Lmo4*

**Fig 7:**
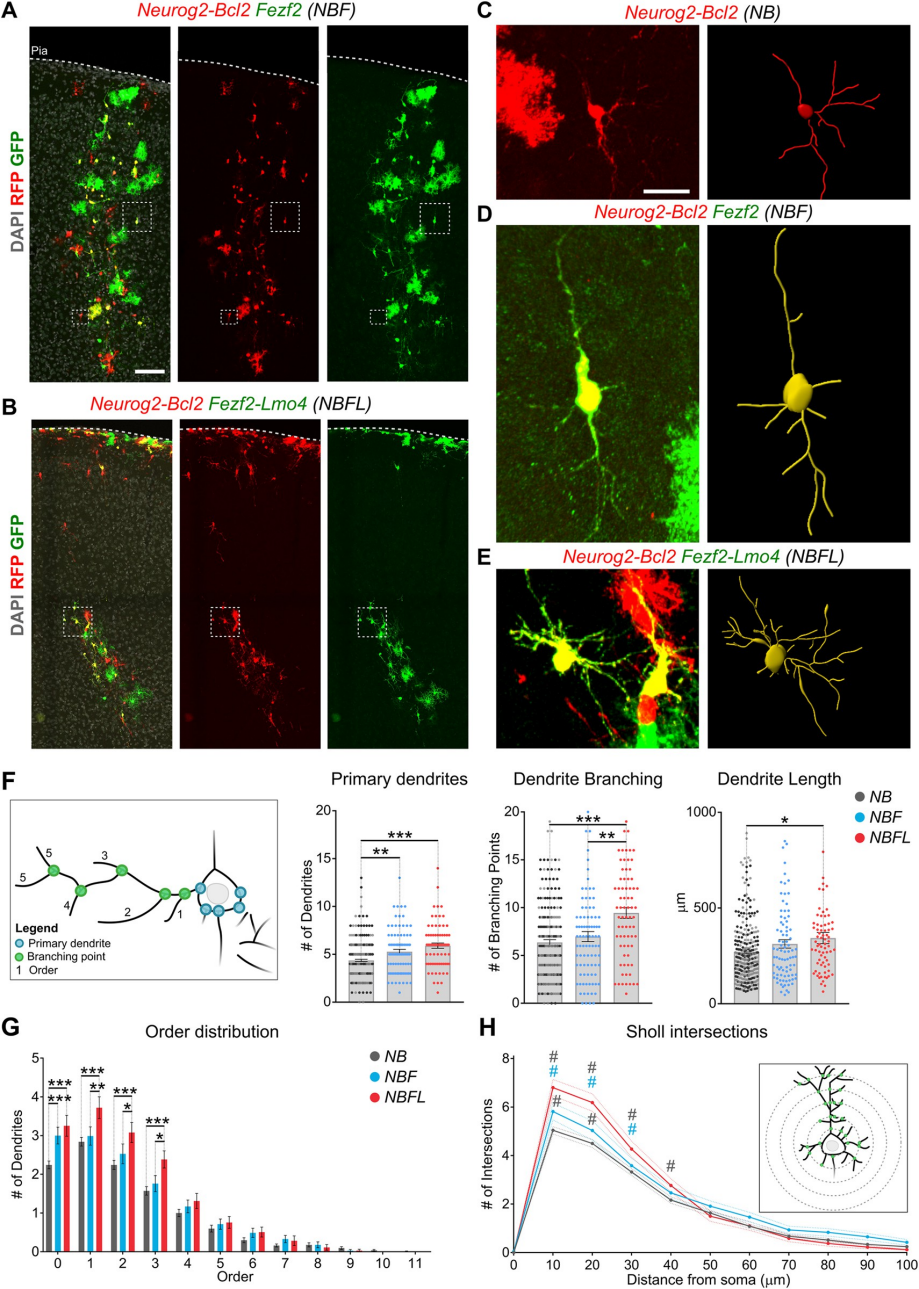
Neurons induced by *NBF* and *NBFL* show increased dendrite complexity. (**A, B**) Representative images of injection sites at 12 dpi. Magnifications and 3D reconstructions **(C–E)** of *NB*, *NBF*, and *NBFL* iNs, respectively. (**F**) Schematic representation of the analyzed morphological parameters. Blue dots represent primary dendrites, defined as filaments originating from the soma; green dots the branching points and numbers indicate the order of each terminal dendrite, defined as the number of branching points the dendrite undergoes from its somatic origin to the terminal tip. Average values (histogram bars) and data distribution (dot plot) of number of primary dendrites, branching points and total dendritic length for *NB* (gray), *NBF* (light blue), and *NBFL* (red) iNs, respectively. While *NBF* iNs only show an increase in primary dendrites compared to *NB* iNs, the addition of *Lmo4* (*NBFL*) induces a stronger effect, reflected by an increased number of primary dendrites, branching points, and increased overall dendritic length. (**G**) Histogram representing the dendrite order distribution in *NB*, *NBF*, and *NBFL* iNs. Compared to *NB* neurons, *NBF* iNs show a significant increase in order 0 dendrites only, and a positive tendency in other categories (1–7). However, the addition of *Lmo4 (NBFL)* leads to an increased number of 0–3 order dendrites and a positive tendency in higher ranks (4–7) dendrites. (**H**) Sholl analysis of *NB* (gray), *NBF* (light blue), and *NBFL* (red) iNs points to increased overall complexity of dendrite arborization in *NBFL* compared to both *NB* and *NBF*. In the inset, a schematic representation of the analysis. Gray hashtags indicate statistical differences between *NB-NBF* and *NB-NBFL* and light blue hashtags between *NBF-NBFL*. Data are represented as mean ± SEM. *N* = 3 animals per condition. (**F, G**) Analyzed cells: *NB*, *n* = 283; *NBF*, *n* = 89; *NBFL*, *n* = 75. *, *p* < 0.05; **, *p* < 0.01; ***, *p* < 0.005. Extended data and statistics are listed in **S6 Data**. GFP, green fluorescent protein.

In light of the role of *Fezf2* promoting not only subcortical axonal projections from DL pyramidal neurons but also their dendritic morphology [50,51], we investigated the dendritic complexity of *NBF* and *NBFL* iNs, by analyzing several distinct morphological parameters of iNs distributed throughout upper and deep cortical layers at 12 dpi (**Fig 7A–7E**). We first evaluated the dendritic arborization geometry and complexity (**Fig 7F**), by assessing the number of primary dendrites and branch points, total dendrite length, and dendrite order distribution (**S6 Data**). This analysis revealed an overall increased dendritic complexity of *NBFL*-reprogrammed glia as compared to *NB* and *NBF* iNs. While the addition of *Fezf2* already increased the number of primary dendrites compared to *NB* iNs, the combined expression of both *Fezf2* and *Lmo4* led to a further rise in the number of primary dendrites and branch points (**Fig 7F**) and resulted on average in improved higher-order dendrites (**Fig 7G**) and increased dendritic length (**Fig 7F**) as compared to *NB* and *NBF* iNs (**S6 Data**).

Finally, we determined Sholl profiles of *NB*, *NBF*, and *NBFL* iNs (**Fig 7H** and **S6 Data**). Notably, the addition of *Fezf2* alone was sufficient to promote basal dendrite complexity (10 to 20 μm from the soma) compared to control *NB* iNs. However, co-expression of *Lmo4* along with *Fezf2* induced a further increase in dendritic complexity (10 to 40 μm from the soma). Together, these data show that the *NBF* and *NBFL* gene combinations not only improve reprogramming efficiency towards a layer V-like neuron identity (**Fig 6**) but also promote the acquisition of more complex morphological features (**Fig 7**), suggesting a more mature state of reprogrammed neurons.

**Table 1:** Qualitative assessment of axonal signals in all experimental conditions.

	Cortex	Corpus callosum	Striatum	Thalamus	Internal capsule	Cerebral peduncle	Spinal cord
E14.5-P7 (Fig 1)							
*cGFP*	+++	+++	+	-	-	-	-
*cFezf2*	+++	++	++	++	++	++	+
*cLmo4* and *cFezf2*	++	-	++	++	++	++	++
E14.5-P7 (Supporting information S2 Fig)							
*cGFP*	+++	+++	+	-	-	-	-
*cLmo4*	+++	+++	-	-	-	-	-
TAM P3—P8 (Fig 5)							
*iGFP*	+++	+++	+	+	+	-	-
*iFezf2*	+++	++	+	+	+	+	+
*iLmo4* and *iFezf2*	+++	+	++	+	++	++	++
TAM P7—P14 (Supporting information S4 Fig)							
*iGFP*	+++	+++	-	-	-	-	-
*iFezf2*	+++	++	-	+	+	+	-
*iLmo4* and *iFezf2*	+++	++	-	+	++	++	+
TAM P10—P21 (Supporting information S5 Fig)							
*iGFP*	+++	++	+	-	-	-	-
*iFezf2*	+++	++	+	-	+	++	-
*iLmo4* and *iFezf2*	+++	++	+	-	++	+	+
TAM P21—P35 (Supporting information S6 Fig)							
*iGFP*	+++	++	+	-	+	-	-
*iFezf2*	+++	++	+	-	+	+	+
*iLmo4* and *iFezf2*	+++	++	+	-	++	++	++

The first column lists the different experimental conditions together with the corresponding figures. The other columns show the intensity levels of the GFP+ or the FLAG+ signals in the specified anatomical structures. Below, the correspondence of the symbols (-, +, ++, +++) and staining levels. The high intensity observed in the cortex (+++) was used as a point of comparison.

## Discussion

### Neuron subclasses reprogramming by *Fezf2* and *Lmo4*

Several reports have shown that proliferating and differentiating postmitotic cell types can be reprogrammed to acquire specific features of a different lineage following the expression of TFs of alternative cell fates (reviewed in [5–7,52]. However, the efficiency of lineage conversion, subtype specificity, and the maturation stage at which neurons are reprogrammed can vary in function of the TF cocktail, different experimental setup, and cell types. By taking advantage of the ability of the TF *Fezf2* to convert callosal projection neurons or cortical stellate neurons to CFuPNs [2,4,21], our data have unveiled a higher reprogramming efficiency of UL neurons than previously reported. We found that the co-expression of *Fezf2* with the co-adaptor *Lmo4* strongly increases the reprogramming competence of UL to acquire a layer V-like identity. Such conversion can rise to 80% when the process occurs in a more conducive environment, such as the motor cortex. This implies that even if *Fezf2* is considered a strong layer V determinant gene that represses alternative cell type-specific genes [51,53], it nevertheless might lack a significant pioneer factor activity on its own, and requires a favorable epigenetic and/or transcriptional context to optimally transactivate its target genes. Indeed, the recently described role of *Fezf2* during astrocyte maturation seems to be dependent on the chromatin state of the cell that expresses it [54]. Although we still do not know the exact mechanisms by which *Lmo4* improves *Fezf2*-mediated conversion, we postulate that *Lmo4* might act as a chromatin modifier of the epigenetic landscape of distinct fate-restricting signals by de-repressing factors that counteract fate changes, as shown in our previous work [31]. Indeed, while the *Ctip2* locus becomes normally silenced in the presence of a multiprotein complex including Satb2, HDAC, and/or Ski [55–57], high levels of *Lmo4* prevent its assembly and release *Ctip2* repression [31]. Although *Lmo4* is considered a late layer V callosal projection neuron marker [28], it is also expressed in subcerebral neurons during early development and promotes layer V projection neuron fate in motor cortex [29]. This suggests that Lmo4 may cooperate with several factors involved in the maturation and refinement of layer V projection neurons. In fact, overexpression of *Lmo4* alone is not sufficient to convert upper- into deep projection neurons (this study), indicating that it needs to work with selector regulators, such as *Fezf2*, to fully accomplish its function. Overall, our findings support a synergistic interaction between *Lmo4* and *Fezf2* in subtype-specific functions during the molecular refinement of neocortical subcerebral versus callosal identity. Based on these findings, we propose that subtype-specific TF-directed reprogramming requires epigenetic regulation to improve efficiency and accuracy.

Besides acquiring DL-like molecular identities, our data show that reprogrammed UL neurons can extend their axons to new targets, despite their ectopic location (i.e., location in ULs). Compared to earlier reports [2,4], the synergy of *Lmo4* and *Fezf2* allows a more robust axonal rewiring of reprogrammed neurons. Already at embryonic stages, high *Fezf2/Lmo4* expression in UL completely abolishes callosal neurons to cross the midline by rewiring them to subcerebral targets, such as the thalamus and cerebral peduncle, in line with a high number of electroporated cells expressing layers V and VI markers. The reprogrammed molecular signature is largely maintained until P35, except for the layer VI marker Darpp32 normally expressed in dopaminoceptive neurons and probably dependent on dopaminergic innervation from the basal ganglia [40,58].

**Fig 8:**
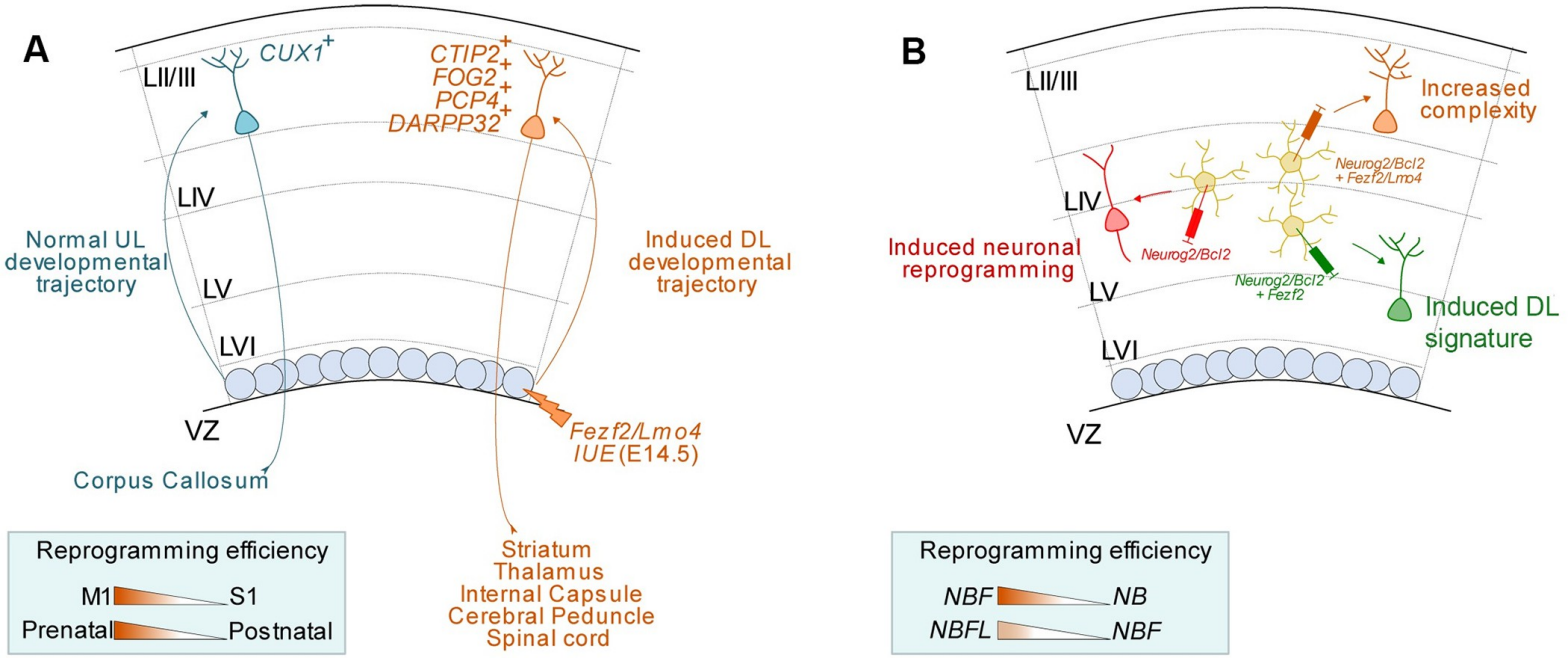
**Model of *Fezf2/Lmo4* synergistic reprogramming effect on pyramidal neurons (A) and glial cells (B). (A)** By electroporating (IUE) *Fezf2* and *Lmo4* in UL neurons normally projecting contralaterally either at embryonic E14.5 or postnatally, these neurons acquire a DL-like signature and tend to project outside of the cortex to subcerebral targets, such as the striatum, thalamus, IC, CP, and/or SC. This lineage conversion is more efficient in the motor (M1) than in the somatosensory (S1) cortex and at prenatal than at postnatal stages. **(B)** Injection of a retrovirus either carrying *Neurog2/Bcl2 (NB), Neurog2/Bcl2* and *Fezf2 (NBF)*, or *Neurog2/Bcl2* and *Fezf2/Lmo4 (NBFL)* into P5 cortical proliferative glial cells leads to increased complexity and a higher proportion of iNs to express the DL marker Ctip2. CP, cerebral peduncle; DL, deep layers; IC, internal capsule; IUE, *in utero* electroporation; SC, spinal cord; UL, upper layers.

Moreover, this study shows that the persistent co-expression of *Fezf2* and *Lmo4* allows a more extended period of postnatal neuronal reprogramming than previously reported with *Fezf2* alone. Induction of *Fezf2/Lmo4* expression at P3 and P7 strongly stimulates the expression of the late layer V marker Pcp4 together with subcerebral axonal rewiring, particularly towards the CP and SC. Although this ability of rewiring becomes progressively reduced as development proceeds, labeled axons can still be detected in the SC upon *Fezf2/Lmo4* induction at P21. The use of the “spaghetti monster” fluorescent protein (smFP), which increases the sensitivity in neurons and notably in axons [46], has helped us in labeling rewired axons. Overall, our study shows a synergistic function of *Lmo4* with *Fezf2* in reprogramming callosal projection neurons into corticofugal, and particularly into subcerebral layer V-like neurons (**Fig 8A**). Whether Lmo4 can enhance the reprogramming capabilities of other TFs in other neurons is still not known but worth investigating further.

### Glia-to-neuron reprogramming by *Fezf2* and *Lmo4*

A fundamental question in the field of direct lineage reprogramming is whether cells that underwent fate conversion can acquire fully mature subtype-specific phenotypes [6]. In fact, in many lineage-reprogramming paradigms, cells adopt a hybrid cell state, thereby interfering with the acquisition of full functionality [59]. Thus, it is of utmost importance to develop strategies to resolve such hybrid states which are likely a consequence of incomplete remodeling of the epigenetic landscape of the cell of origin. In this study, after having noted strong DL promoting activity in early and late postmitotic cortical neurons, we examined the effect of co-expressing the terminal selector gene *Fezf2* and the transcriptional adaptor *Lmo4* together with a well-established reprogramming factor cocktail of the proneural gene *Neurog2* and the anti-cell death regulator *Bcl2* [15,18]. Towards this, we utilized a reprogramming paradigm that allows for selective targeting of proliferative glia (i.e., approximately 2/3 astroglia and 1/3 oligodendrocyte progenitor cells) in the early postnatal cerebral cortex by retroviruses encoding reprogramming factors [15]. We found that consistent with its role as terminal selector gene during cortical development [51,53], *Fezf2* enhanced expression of the cortical layer V marker Ctip2 in neurons induced by *Neurog2/Bcl2* and *Fezf2* alone or in combination with *Lmo4*, which appeared to exert little neurogenic reprogramming activity on its own. This effect was most accentuated in cortical layer V where Ctip2 neurons are most abundant (**Fig 8B**). These data could point to the intriguing possibility that the local environment exerts significant influence on the molecular maturation of iNs via its layer-specific neuronal and glial signaling compartments. Contrary to what we have observed in the neuron-to-neuron reprogramming paradigm, co-expression of *Lmo4* did not overtly enhance the deep-layer fate-inducing action of *Fezf2*. However, we noticed increased dendritic complexity of iNs expressing the full reprogramming cocktail (*NBFL*). These data argue that *Fezf2* and *Fezf2/Lmo4* regulate distinct aspects of neuronal subtype identity in iNs. Some targets, e.g., the *Ctip2* gene may be sufficiently accessible in the context of *Neurog2*/*Bcl2*-mediated reprogramming to become activated by *Fezf2*. In contrast, developing a complex dendritic arborization is likely to be dependent on several molecular players, besides *Fezf2* [50], and hence be more vulnerable to incomplete epigenetic remodeling, therefore, requiring extra machinery such as that provided, for example, by *Lmo4*. Finally, our morphological reconstructions could not assess whether iNs would extend axonal projections to subcortical targets such as those observed in the neuron-to-neuron reprogramming paradigm. Forming successful connections with target cells is very likely to be of prime importance for iNs achieving full maturation in terms of molecular identity and physiological properties. While our reprogramming paradigm contributes to deepening our understanding of cell fate boundaries and how these can be overcome, reprogrammed cells likely face unique challenges for incorporation in preexisting cortical circuits, and we are still in a stage where these need to be understood to improve reprogrammed neurons survival and integration. We do need to realize that reprogramming is a challenging process, especially in an unlesioned environment. Overall, our findings presented here provide an important molecular entry point into further tuning glia-to-neuron reprogramming toward generating iNs that resemble endogenous neurons in connectivity and functionality.

### Limitations of the study

The findings of this study must be seen in light of some limitations. First, we conducted the analysis of the postnatal glia-to-neuron *in vivo* reprogramming experiment in an unlesioned P5 brain only at 12 dpi, based on previous evidence of retrovirus-mediated glia-to-neuron reprogramming and acquisition of neuronal subtype-specific hallmarks within 1 to 3 weeks [12,15,18]. At this early time point, the reprogrammed neurons did not reach a fully mature state, and this is largely reflected in their morphology, which is different from the morphology of endogenous cortical neurons. At this stage, reprogrammed neurons appear smaller in size, with a smaller body size, and no spine-like structures were found. This is consistent with earlier findings of the presence of spine-like structures only from later reprogramming stages in Neurog2-Bcl2 reprogrammed glia in the adult lesioned cortex [18].

Second, our study is represented by the technical challenges of reliably quantifying axons and tracts upon IUE. In this study, we prioritized a strong and extensive molecular characterization of our IUE reprogrammed neurons, which required extensive tissue sectioning, 2D immunostaining, and high-resolution imaging. However, this limited the power of our tract and axon analysis, which we decided to include solely as a merely qualitative assessment. To better investigate connectivity changes in IUE experiments, a more extensive analysis would be to rely on more advanced technologies (i.e., 3D imaging or spatial alignment to a common 3D reference atlas). However, considering the high number of conditions evaluated in this study (4 distinct genetic conditions for 5 time points), we privileged a high-throughput experimental setup for this work, rather than including a comprehensive connectivity analysis, which would instead benefit from being published as a stand-alone study.

## Methods

### Ethics statement

All experiments were conducted in accordance with the French and German Animal Welfare Act and European guidelines for the use of experimental animals, using protocols approved by the French Ministry of Education, Research and Innovation (reference # APAFIS#l 8019–2018112919027679 v4) and the local ethics committee (CIEPAL NCE/2019–548, Nice), and the Rhineland-Palatinate State Authority (permit number 23 177 07-G-15-1-031). All experiments were conducted on RjHan:NMRI mice or C56Bl6/J mice (retroviral injection experiments) obtained from *Janvier Labs*. Upon IUE only (**Figs 1–5**) or followed by tamoxifen injection (**Figs 4** and **5**) or retroviral injection (**Figs 6** and **7**), both male and female animals were used for the analyses. Female and male RjHan:NMRI or C56Bl6/J mice were put in mating in the evening, and midday of the day of the observed vaginal plug was considered as embryonic day 0.5 (E0.5).

### Plasmids

For embryonic reprogramming at E14.5, the *pCdk5r-Lmo4-IRES-GFP*, *pCdk5r-Fezf2-IRES-GFP*, and *pCdk5r-IRES-GFP* plasmids were used. *pCdk5r-Fezf2-IRES-GFP* and *pCdk5r-IRES-GFP* were donated by the lab of Paola Arlotta [2], while *pCdk5r-Lmo4-IRES-GFP* was generated in the lab (Harb and colleagues). All plasmids were tested for correct expression (**S1 Fig**). To induce the expression of *Lmo4* and *Fezf2* at postnatal stages upon IUE, *Fezf2* and *Lmo4 cDNAs* were subcloned into the *pCAG-fl-mutCherry-fl-IRES-EGFP* vector [44] by substituting the *mutCherry* with *Fezf2* or *Lmo4*, resulting in *pCAG-Ind-GFP (iGFP)*, *pCAG-Ind-Fezf2 (iFezf2)*, and *pCAG-Ind-Lmo4 (iLmo4*). The plasmids consist of a *stop* sequence flanked by *loxP* sites in the same directional orientation as the inserted cDNAs and followed by an IRES-EGFP sequence to allow EGFP reporter expression. Correct expression of the genes of interest was then elicited via a Tamoxifen-dependent Cre recombination (**S3 Fig**). The *pCAG-CreERT2* plasmid is commercially available from *Addgene* (#13777). Since the GFP of the inducible *Lmo4* and *Fezf2* plasmids was not appropriate for following reporter expression into secondary axons and collaterals of electroporated cells, the *pCAG-smFP_FLAG (Addgene* #59756) was added to the reprogramming factors to facilitate axonal tract tracing (**Figs 5** and **S4–S6**).

### *In utero* electroporation (IUE)

The experimental procedure for IUE was performed according to the protocol of Saito and Nakatsuji [60] with the following modifications: endotoxin-free plasmids were diluted in TE-Buffer (Qiagen, #1018499) until a cumulative concentration of 1 μg/μl in a final volume of 20 μl, including 1× Fast Green FCF (Sigma-Aldrich, #F7252). Pregnant females of 14.5 days were deeply anesthetized via intraperitoneal (i.p.) injection of 250 μl to 350 μl of Tiletamine-Zolazepam-Xylazine-Buprenorphine, shaved on the upper abdomen, and cleaned with disinfectant (Pierre Fabre, #716431). After a lateral laparotomy was performed, the uterus was exposed by carefully pulling with ring forceps. Microinjection of the DNA mix was performed using capillaries (Harvard-Apparatus, #30–0016), produced with a micropipette puller (Sutter Instrument, Model P-1000; Parameters: Heat 459, Pull 60, Vel 75, Time/del 100, Pressure 200), and cut open at approximately 60 μm from the tip. The capillaries were inserted into a holder connected to a microinjector (Eppendorf FemtoJet 5274 V2.02; Parameters: Pi[hPA] 100–300, ti[s] 0.7, Pc[hPA] 7) and approximately 1 μl of the plasmid mix was injected into one of the 2 lateral ventricles. To direct the DNA mix to the region of interest, as well as open pores on the cell membrane and facilitate DNA intake, tweezer electrodes (3 mm, Nepagene, #CUY650P3) were placed onto the uterine wall, with the plus node on the premature M1 or S1 area underneath, to apply square electric pulses (without poring pulse; Transfer pulse Voltage: 37, Pulse length: 50 ms, Pulse interval: 999 ms, Number of pulses: 4, Decay rate: 5%) via an electroporator device (Nepagene Superelectroporator, Nepa21 TypeII). The uterus was subsequently repositioned into the abdomen and the incision was sewed up with sutures (Péters Surgical; 6/0 #87002F for peritoneum and 5/0 #87001F for skin). Approximately 120 μl of the anti-inflammatory Meloxicam (Metacam) and 200 μl of the antibiotic Gentamicin (1 mg/ml, Sigma #G1272) were subcutaneously injected to prevent inflammation. Electroporated mice were then housed in a solitary ventilated black box for 24 h after the surgery to recover, before being then transferred to the husbandry room.

### Tamoxifen injection

To induce expression of the CRE-recombinase from the *pCAG-CRE-ER^T2^* plasmid, 4-hydroxy tamoxifen (TAM) was administered at the desired developmental stage. Briefly, 10 ml of 10 mg/ml TAM was prepared by dissolving 100 mg TAM (Sigma, #T5648-1G) in 500 μl ethanol absolute in an Eppendorf tube. The solution was stirred on a rotator for 20 min at RT, before diluting it with 9.5 ml of corn oil (Sigma, #C8267). Then, the mix was thoroughly vortexed and put back on a rotator until complete dissolution and 1 ml aliquots were prepared and stored at −20°C until usage. Mice received a single administration of tamoxifen via subcutaneous injections under the neck. Injected volumes adapted to their age: P3-P7, 50 ml; P10-P14, 100 ml; P21, 150 ml.

### Retroviral constructs and retrovirus injections

MMLV-based retroviral vectors were used for overexpression of the reprogramming factors in the cerebral cortex of postnatal day 5 (P5) mice [49]. The following constructs were used: *CAG-mNeurog2-T2A-hBcl2-IRES-DsRed* [15], *CAG-Fezf2-IRES-eGFP* and *CAG-Fezf2-P2A-Lmo4-IRES-eGFP*, which were cloned by inserting the cDNAs under the control of the chicken β-actin promoter with a cytomegalovirus enhancer (pCAG) and an enhanced GFP or DsRed reporter cloned behind an Internal Ribosome Entry Site (IRES) to identify transduced cells. Vesicular Stomatitis Virus Glycoprotein (VSV-G)-pseudotyped retroviral particles were produced using gpg helper-free packaging cells [61]. Viral titers used were in the range of 10^7^ TU/ml, as measured by transduction of HEK293 cells. Retroviral particles were injected into the cerebral cortex of male and female P5 WT C57Bl6/J mice kept with their mother purchased from Janvier Labs. Mice were housed in Polycarbonate Type II cages (350 cm2) in a 12:12 h light-dark cycle and were provided with food and water ad libitum. All efforts were made to reduce the number of animals and their suffering. A subcutaneous injection of Carprofen (Rimadyl, Zoetis, 4 mg/kg of body weight, in 0.9% NaCl [Amresco]) was made prior to the surgery and anesthesia was induced by i.p. injection of a solution of 0.5 mg/kg Medetomidin (Pfizer), 5 mg/kg Midazolam (Hameln), and 0.025 mg/kg Fentanyl (Albrecht) in 0.9% NaCl. A small skin incision was made above the somatosensory cortex and the skull was minimally opened using a needle, and 0.5 to 1 μl of retroviral suspension was delivered into the cerebral cortex using pulled glass capillaries (20 μm tip diameter, Hirschmann, 9600105). After injection, the capillary was carefully removed, and the wound was closed with surgical glue (3M Vetbond, NC0304169). Animals then received an i.p. injection of a solution of 2.5 mg/kg Atipamezol (Pfizer), 0.5 mg/kg Flumazenil (Hameln), and 0.1 mg/Kg Buprenorphin (RB Pharmaceutials) in 0.9% NaCl. Pups were kept on a warm plate (37°C) before returning them to their mother and their recovery state was scored daily for a week after the surgery.

### Histology and immunohistochemistry

At the planned developmental stage, *IUE* mice were intracardially perfused with phosphate buffer saline (PBS, 15 ml) and varying amounts of 4% paraformaldehyde (PFA, Sigma-Aldrich, #P6148), according to the animal age (15 ml for pups and young animals until P21, 30 ml for adults). Afterwards, the head was cut and dissected. After checking for successfully electroporated brains (GFP-positive) under an epifluorescence microscope, the tissues were post-fixated in 4% PFA for 2 h at room temperature (RT). Then, brains were washed 1 time in PBS and embedded in 4% Select Agar (Sigma-Aldrich, #A5054) in PBS. All brains were cut coronally in 100 μm-thick vibratome sections (Leica VT 1000S; Speed: 7, Frequency 7) and preserved in PBS-Azide (0.05%) until further processing. For immunostaining, floating slices were incubated in 1 ml blocking buffer (10% Goat serum [Life Technologies, #16210064], 3% BSA [Sigma-Andrich, #A3294], 0.03% Triton-X100 [Sigma-Aldrich, #T8787] in PBS 1×) per well overnight (ON) at 4°C, gently rocking. Then, primary antibodies were diluted in 500 μl antibody buffer (3% Goat serum, 3% BSA, 0.03% Triton-X100 in PBS 1×) per well for 2 days at 4°C. Subsequently, slices were washed 3 times for 10’ at RT and 2 times for 1 h. Secondary antibodies were diluted 1:400 in antibody buffer and 500 μl were added to each well and incubated ON at 4°C. Slices were washed again, as described before, then incubated with 500 μl of DAPI (diluted 1:10,000 in PBS—Invitrogen, #H3570) at RT for 5’ before mounted on glass microscope slides (Thermo Scientific, #J1800AMNZ) with 200 μl of Mowiol mounting medium and enclosed with a cover slip. In the brains electroporated with the *cGFP*, *cFezf2*, and *cLmo4* plasmids, the GFP signal was enhanced using an anti-GFP antibody, whereas in the brains electroporated with the *iGFP*, *iFezf2*, *iLmo4* together with the *pCAG-smFP_FLAG* plasmids, only the anti-FLAG antibody was used to visualize axonal projections (see Supporting information S1 Table for antibody details).

Retrovirally injected tissue was processed as follows. After terminal anesthesia with a solution of 120 mg/kg Ketamine (Zoetis) and 16 mg/kg Xylazine (Bayer) (in 0.9% NaCl, i.p.), mice were transcardially perfused with pre-warmed 0.9% NaCl followed by ice-cold 4% PFA (Sigma, P6148). Brains were post-fixed overnight in PFA 4% at 4°C and sliced coronally (40 μm thick sections) using a vibratome (Microm HM650V, Thermo Scientific). Brain sections were then stored at −20°C in a cryoprotective solution (20% glucose [Sigma, G8270], 40% ethylene glycol [Sigma, 324558], 0.025% sodium azide [Sigma, S2202], in 0.5 X phosphate buffer [15 mM Na2HPO4·12H2O [Merck, 10039-32-4]; 16mM NaH2PO4 ·2H2O [Merck, 13472-35-0]; pH 7.4]). For immunohistochemistry, free-floating sections were washed 3 times for 15 min with 1× TBS (50 mM Tris [Invitrogen, 15504–020]; 150 mM NaCl [Amresco, 0241]; pH7.6), incubated for 90 min in blocking solution (5% Donkey Serum [Sigma, S30]; 0.3% Triton X-100; 1× TBS) and then with primary antibodies (diluted in blocking solution) for 2 to 3 h at RT and overnight at 4°C. After 3 washes with 1× TBS, slices were incubated with secondary antibodies (diluted blocking solution) for 90 min at RT. Sections were washed twice with 1× TBS, incubated with DAPI (in 1× TBS, Sigma, D8417) for 5 to 7 min at RT, washed 3 times with 1× TBS and 2 times with 1× phosphate buffer (30 mM Na2HPO4·12H2O [Merck, 10039-32-4]; 33 mM NaH2PO4 ·2H2O [Merck, 13472-35-0]; pH 7.4). Finally, sections were mounted on Superfrost (Thermo Fisher Scientific) microscope slides and covered with coverglasses using Prolong Gold (Invitrogen, #P36930). For the complete list of primary and secondary antibodies, see Supporting information S1 Table.

### Microscopic imaging and image analysis

For whole brains and tract tracing imaging, mosaic microscopic images were acquired using an Axio Imager M2 epifluorescence microscope (Carl Zeiss Microscopy GmbH, Jena, Germany) equipped with a halogen lamp, MCU 2008 motorized stage, and an EC Plan-Neofluar 10×/0.30 and an AxioCam MRm camera. ZEN blue software was used for imaging and automatic stitching. For molecular analyses, imaging was performed using a Zeiss 710 confocal microscope equipped with a 405 nm diode, an argon ion, a 561 nm DPSS, and a 647 HeNe lasers. For molecular studies as well as morphological characterization of retrovirus-infected glial cells, Z-stacks of fixed cortical sections were imaged using a LD-LCI Plan-Apo 25×/0.8 NA. Images from immunostaining experiments were analyzed by using Fiji-ImageJ Software. Each immunofluorescent slice was carefully checked for the right neocortical area (M1 or S1) and their respective anatomy using Allen Brain Atlas or Atlas of the Developing Mouse Brain [62]. Experiments were given random numbers to avoid bias in the analysis. Four layers of confocal images were stacked (2 μm × 4) and the total number of GFP-positive cells counted. Then, markers were screened for co-localization with GFP-positive cells using orthogonal view for 3D presentation. Then, the sum of double GFP-marker positive cells was divided by the number of total GFP-positive cells to assess the prevalence of each marker on the total GFP population. Serial z-stacks images of retrovirally transduced tissue sections were acquired with a TCS SP5 (Leica) confocal microscope (Institute of Molecular Biology, Mainz, Germany) equipped with 4 lasers (405 Diode, Argon, HeNe 543, HeNe 633) using a 20× dry objective (NA 0.7) or a 40× oil objective (NA 1.3), or with an Axio Imager.M2 fluorescent microscope equipped with an ApoTome (Zeiss) using a 20× dry objective (NA 0.7). Analysis of DCX, NeuN, and Ctip2 expression in retrovirally transduced cells was performed in ImageJ using the Cell Counter tool. For calculation of the depth position of the cells relative to the cortical surface, the value of the y-position (depth axis) of the cortical surface was measured with 80 μm intervals along the x-axis (latero-medial axis). Each of these values thereby defined the y-position of the cortical surface at the center of 80-μm wide virtual horizontal columns. For each cell, the y-position of the cortical surface of the virtual column it was located in, was subtracted to the measured value of the y-position of the cell.

Morphological features of retrovirally transduced cells were analyzed using IMARIS software. Cell arborization was reconstructed in a semi-automatic manner via the filament tracer module. An automatic length measuring tool was used to calculate the total dendritic length and the basal dendritic length. Primary dendrites (protrusion originating from the soma), branching points, and branching point orders (integer values equivalent to the number of branching points a dendrite undergoes from its somatic origin to the terminal tip) were manually quantified. An automatic detection of Sholl intersections was used to compute Sholl profiles for every individual cell. Sholl intersections were identified as the number of dendrite intersections for concentric spheres of increasing radius and having as a center the centroid of the cell body. Distance between radii was set at 10 μm.

### Statistical analysis

Graphs and statistical analysis have been performed with Graphpad Prism 7. The results of the molecular analyses (**Figs 1–4**) are represented as percentages and error bars were calculated as standard error of the mean (SEM). For comparisons between plasmids, one-way ANOVA was applied, followed by Tukey post hoc test. For grouped comparisons between areas and plasmids, two-way ANOVA followed by Tukey or Sidak post hoc test was used. For each experiment, between 3 and 4 sections per brain were imaged in the somatosensory or motor cortex of electroporated brains. Quantification of GFP+ cells expressing a specific combination of markers were averaged for each brain, and then pooled together with other 2 independent brains, each of which had 3 sections. In total, 9 sections (3 per brain) were quantified to generate the data shown in the histograms and data sets. “n” refers to the number of animals analyzed. Data sets from the morphological characterization of retrovirally transduced cells (**Fig 7**) were tested for normality (Kolmogorov–Smirnov) or homoscedasticity (Levene test) before performing parametric (Student *t* test, one-way or two-way ANOVA followed by Bonferroni’s post hoc test) or nonparametric (Welch’s unequal variances *t* test or Mann–Whitney test) tests used to determine *P*-values. Retrovirus-injection data are represented as mean ± SD. The number of independent experiments (n) and the number of cells analyzed is indicated in the figure legend. *P*-values are included in the figure. Graphs and statistical analysis were performed in GraphPad Prism7 and SPSS Statistics 23 V5 (IBM), respectively. Shapiro–Wilk test was used for testing normality of the distribution of the data.

For normally distributed data, Levene test was used to test homogeneity of variances and one-way ANOVA (in the case of equal variances) or Welch-ANOVA (in the case of non-equal variances) followed by a Tukey post hoc test was used for comparison of means. For non-normally distributed data, the Kruskal–Wallis test by rank was used for comparison of means. All original data are listed in S1 to S6 data sets.

## Supporting information

S1 Fig *cFezf2* and *cLmo4* are properly expressed in GFP+ electroporated cells. **(A)** Schematic representation of the experimental design. *cLmo4 (cL)* and/or *cFezf2 (cF)* plasmids were electroporated into E14.5 somatosensory (S1) embryonic cortices. Brains were collected at P7. **(B)** Validation of the correct expression of Fezf2 and Lmo4 proteins by immunofluorescence of electroporated GFP+ cells. Note that almost 90% of GFP+ cells do express Lmo4 and Fezf2 (arrowheads point to triple positive, arrows to single or double positive cells). (**C**) Double staining of layers V and VI markers with Ctip2, a well-described layer V marker in the somatosensory (S1) cortex of WT brains. Percentages shown as mean ± SEM indicate the degree of co-localization of Fog2, Pcp4, and Darpp32 with high or low expression of Ctip2 in layers V and VI. All individual data are listed in S1 Data.
(PDF)

S2 Fig Overexpression of *cLmo4* fails to lead to any lineage conversion and axonal rewiring. **(A)** Schematic representation of the experimental procedure and vectors. *cGFP* and *cLmo4 (cL)* plasmids were electroporated into E14.5 somatosensory (S1) embryonic cortices. Brains were collected at P7. **(B)** Immunofluorescence (IF) of GFP on a coronal slice of an electroporated brain confirms the expected laminar localization (layers II–IV) of electroporated GFP+ cells. (**C**) Percentage of S1-electroporated UL neurons expressing UL vs. DL markers. (**D**) Representative images of Cux1, Ctip2, Fog2, Pcp4, and Darpp32 IF staining in electroporated brains. Full and empty arrowheads respectively indicate whether GFP+ cell co-express or not, respectively, the marker. (**E**) Tract tracing of upper-layer GFP+ axons upon electroporation of *cGFP* and *cLmo4* in P7 brains. No particular changes between *cGFP-* and *cLmo4*-electroporated brains have been detected. Scale bars: B = 1,000 μm (left, macro image) and 200 μm (right, magnified image); D = 20μm; E = 1,000 μm (top row, macro images), 200 μm (magnified images). Results are expressed as mean ± SEM. Two-way ANOVA with Tukey’s post hoc correction was used for statistical analysis, **p < 0.01, ***p < 0.0001. *n* = 3 brains for each plasmid. CC, corpus callosum; CP, cerebral peduncle; IC, internal capsule; SC, spinal cord; Str, striatum; Th, thalamus. All individual data and statistics are listed in S1 Data. See also Table 1.
(PDF)

S3 Fig *iFezf2* and *iLmo4* are properly expressed in induced electroporated brains. (**A**) Schematic representation of the experimental procedure and vectors used in this experiment. *iFezf2 (iF)* and *iLmo4 (iL)* were electroporated into E14.5 somatosensory (S1) cortices. Gene expression was induced at P3 by tamoxifen (TAM) subcutaneous injection. Brains were collected at P8. (**B**) Immunofluorescence (IF) of GFP, upper-layer marker Cux1 and deep-layer V marker Ctip2 on a coronal slice of an electroporated brain. White box indicates magnification image on the right side. **(C)** Validation of the correct expression of *Fezf2* and *Lmo4* proteins by IF of TAM-induced brains. Note that almost all GFP+ cells express *Lmo4* and/or *Fezf2*. Below, confocal images of high-magnification panels showing 3D reconstructions of double staining. Side bars represent projections along the x–z axes (right) and the y–z axes (below). (**D**) No GFP expression is detected in NO-TAM-induced brains co-electroporated with mCherry (in red). Scale bars: B = 1,000 μm (left, macro image) and 200 μm (right, magnified image); C, D = 20 μm.
(PDF)

S4 Fig P7 induction of *Fezf2* and *Lmo4* expression can partially change upper-layer axonal projections toward subcerebral targets. (**A**) Schematic representation of the experimental procedure and vectors. *iGFP, iFezf2 (iF)*, or *iFezf2* and *iLmo4 (iF+iL)* together with *pCAG-CRE-ER^T2^* were electroporated into E14.5 somatosensory (S1) cortices. The *smFP-Flag* reporter plasmid was co-electroporated to facilitate axon tracing. Gene expression was induced at P7 by tamoxifen subcutaneous injection. Brains were collected at P14. (**B**) Tract tracing of upper-layer FLAG+ axons upon electroporation of *iGFP, iF* or, *iF+iL* vectors. Full and empty arrows indicate presence or absence of axons, respectively. FLAG+ axons were found crossing the corpus callosum (CC) in all conditions, but they reached the thalamus (Th), internal capsule (IC), and cerebral peduncle (CP) only in *iF* and *iF+iL* conditions; axons were clearly detected in the spinal cord (SC) of *iF+iL* brains. White boxes indicate regions magnified in the panels below or aside. Scale bars: B = 1,000 μm (macro images) and 20 μm (magnification images). *n* = 3 brains for each plasmid. Str, striatum. See also Table 1.
(PDF)

S5 Fig P10 induction of double *Fezf2* and *Lmo4* expression can partially change upper-layer axonal projections toward subcerebral targets. (**A**) Schematic representation of the experimental procedure and vectors. *iGFP, iFezf2 (iF)* or *iFezf2*, and *iLmo4 (iF+iL)* together with *pCAG-CRE-ER^T2^* were electroporated into E14.5 somatosensory (S1) cortices. smFP-Flag reporter plasmid was co-electroporated to facilitate axon tracing. Gene expression was induced at P10 by tamoxifen subcutaneous injection. Brains were collected at P21. (**B**) Tract tracing of upper-layer FLAG+ axons upon electroporation of *iGFP, iF,* or *iF+iL* vectors. Full and empty arrows indicate the presence or absence of FLAG+ axons, respectively. Axons were found crossing the corpus callosum (CC) and reaching the striatum (Str) in all conditions, but their presence was detected in the internal capsule (IC) and cerebral peduncle (CP) only in *iF*- and *iF+iL*-electroporated brains. Although big bundles of axons can be observed in the CP of *iF*-electroporated brains, no obvious projections seem to reach the spinal cord (SC), differently from *iF+iL*-electroporated brains in which few dispersed axons ultimately reach the SC. White boxes indicate regions magnified in the panels below or aside. Scale bars: B = 1,000 μm (macro images) and 20 μm (magnification images). *n* = 3 brains for each plasmid. See also Table 1.
(PDF)

S6 Fig P21 induction of single *Fezf2* and double *Fezf2* and *Lmo4* expression can still partially change upper-layer axonal projections toward subcerebral targets. (**A**) Schematic representation of the experimental procedure and vectors. *iGFP, iFezf2 (iF)* or *iFezf2*, and *iLmo4 (iF+iL)* together with *pCAG-CRE-ER^T2^* were electroporated into E14.5 somatosensory (S1) cortices. smFP-Flag reporter plasmid was co-electroporated to facilitate axon tracing. Gene expression was induced at P21 by tamoxifen subcutaneous injection. Brains were collected at P35. (**B**) Tract tracing of upper-layer FLAG+ axons upon electroporation of *iGFP*, *iF*, or *iF+iL* vectors. Full and empty arrows indicate the presence or absence of FLAG+ axons, respectively. Axons were found crossing the corpus callosum (CC) and reaching the striatum (Str) and internal capsule (IC) in all conditions. However, labeled axons were only detected in the cerebral peduncle (CP) and spinal cord (SC) of *iF*- and *iF+iL*-electroporated brains. White boxes indicate regions magnified in the panels below or aside. Scale bars: B = 1,000 μm (macro images) and 20 μm (magnification images). *n* = 3 brains for each plasmid. See also Table 1.
(PDF)

S7 Fig *NBF* and *NBFL* induce glia-to-neuron reprogramming in all cortical layers. (**A**) Analysis at 12 dpi of DCX-expression in *NBF*- and *NBFL*-cells with regards to their depth position (relative to the cortical surface) shows that reprogramming is induced at similar rates in all cortical layers. Apparent local accumulation of iNs in the first 200 μm below the cortical surface reflects higher numbers of transduced cells at this depth, but not a difference in the reprogramming efficiency (i.e., percentage of DCX-expressing cells among transduced cells). In the graphs, each dot represents a cell. *n* = 3 brains/retrovirus combination, 2,827 cells anaylzed. Images illustrate cell distribution in the cortex from the surface to deep-layers. All individual data are listed in S5 Data.
(PDF)

S1 Data Summary of statistical analysis and results illustrated in Figs 1 and S2C. Highlighted in blue, comparisons that produced statistically significant *P*-values.
(XLSX)

S2 Data Summary of statistical analysis and results illustrated in Fig 2. Highlighted in blue, comparisons that produced statistically significant *P*-values.
(XLSX)

S3 Data Summary of statistical analysis and results illustrated in Fig 3. Highlighted in blue, comparisons that produced statistically significant *P*-values.
(XLSX)

S4 Data Summary of statistical analysis and results illustrated in Fig 4. Highlighted in blue, comparisons that produced statistically significant *P*-values.
(XLSX)

S5 Data Summary of statistical analysis and results illustrated in Fig 6. Highlighted in blue, comparisons that produced statistically significant *P*-values.
(XLSX)

S6 Data Summary of statistical analysis and results illustrated in Fig 7. Highlighted in blue, comparisons that produced statistically significant *P*-values.
(XLSX)

S1 Table Complete list of antibodies used in this study. (PDF)

## Abbreviations

CC: corpus callosum
CFuPN: corticofugal projection neuron
CP: cerebral peduncle
DL: deep layers
GFP: green fluorescent protein
IC: internal capsule
IUE: *in utero* electroporation
MMLV: Moloney murine leukemia virus
ROS: reactive oxygen species
RT: room temperature
SC: spinal cord
smFP: spaghetti monster fluorescent protein
TF: transcription factor
UL: upper layers
