# Supplementary material for: Lmo4 synergizes with Fezf2 to promote direct in vivo reprogramming of upper layer cortical neurons and cortical glia towards deep-layer neuron identities
Source: PLoS Biol. 2023 Aug 8;21(8):e3002237. doi: 10.1371/journal.pbio.3002237 (PMC10409279; doi:10.1371/journal.pbio.3002237)
Supplement: S1 Fig — (A) Schematic representation of the experimental design. cLmo4 (cL) and/or cFezf2 (cF) plasmids were electroporated into E14.5 somatosensory (S1) embryonic cortices. Brains were collected at P7. (B) Validation of the correct expression of Fezf2 and Lmo4 proteins by immunofluorescence of electroporated GFP+ cells. Note that almost 90% of GFP+ cells do express Lmo4 and Fezf2 (arrowheads point to triple positive, arrows to single or double positive cells). (C) Double staining of layers V and VI markers with Ctip2, a well-described layer V marker in the somatosensory (S1) cortex of WT brains. Percentages shown as mean ± SEM indicate the degree of co-localization of Fog2, Pcp4, and Darpp32 with high or low expression of Ctip2 in layers V and VI. All individual data are listed in S1 Data. (PDF) [file pbio.3002237.s001.pdf]

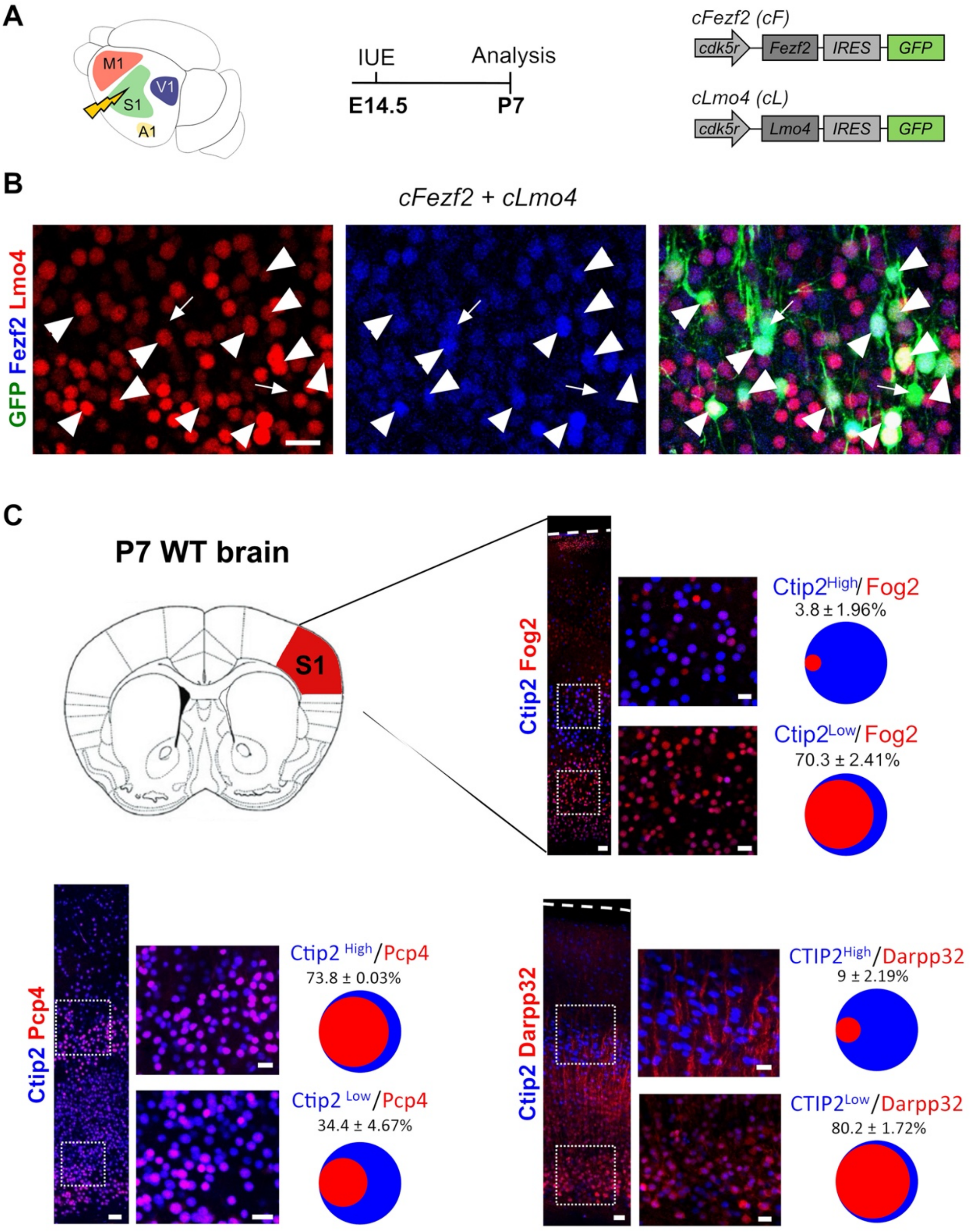

S1 Fig: cFezf2 and cLmo4 are properly expressed in GFP+ electroporated cells. (A) *Schematic representation of the experimental design. cLmo4 (cL) and cFezf2 (cF) plasmids were electroporated into E14.5 somatosensory (S1) embryonic cortices. Brains were collected at P7.* ***(B)*** *Validation of the correct expression of Fezf2 and Lmo4 proteins by immunofluorescence of electroporated GFP+ cells. Note that almost all GFP+ cells do express Lmo4 and Fezf2 (arrowheads point to triple-positive, arrows to single or double-positive cells).* ***(C)*** *Double staining of layers V and VI markers with Ctip2, a well-described layer V marker in the somatosensory (S1) cortex of WT brains. Percentages shown as mean ± s.e.m. indicate the degree of co-localization of Fog2, Pcp4 and Darpp32 with high or low expression of Ctip2 in layers V and VI. All individual data are listed in S1 Data.*
