# Supplementary material for: Lmo4 synergizes with Fezf2 to promote direct in vivo reprogramming of upper layer cortical neurons and cortical glia towards deep-layer neuron identities
Source: PLoS Biol. 2023 Aug 8;21(8):e3002237. doi: 10.1371/journal.pbio.3002237 (PMC10409279; doi:10.1371/journal.pbio.3002237)
Supplement: S2 Fig — (A) Schematic representation of the experimental procedure and vectors. cGFP and cLmo4 (cL) plasmids were electroporated into E14.5 somatosensory (S1) embryonic cortices. Brains were collected at P7. (B) Immunofluorescence (IF) of GFP on a coronal slice of an electroporated brain confirms the expected laminar localization (layers II–IV) of electroporated GFP+ cells. (C) Percentage of S1-electroporated UL neurons expressing UL vs. DL markers. (D) Representative images of Cux1, Ctip2, Fog2, Pcp4, and Darpp32 IF staining in electroporated brains. Full and empty arrowheads respectively indicate whether GFP+ cell co-express or not, respectively, the marker. (E) Tract tracing of upper-layer GFP+ axons upon electroporation of cGFP and cLmo4 in P7 brains. No particular changes between cGFP- and cLmo4-electroporated brains have been detected. Scale bars: B = 1,000 μm (left, macro image) and 200 μm (right, magnified image); D = 20μm; E = 1,000 μm (top row, macro images), 200 μm (magnified images). Results are expressed as mean ± SEM. Two-way ANOVA with Tukey’s post hoc correction was used for statistical analysis, **p < 0.01, ***p < 0.0001. n = 3 brains for each plasmid. CC, corpus callosum; CP, cerebral peduncle; IC, internal capsule; SC, spinal cord; Str, striatum; Th, thalamus. All individual data and statistics are listed in S1 Data. See also Table 1. (PDF) [file pbio.3002237.s002.pdf]

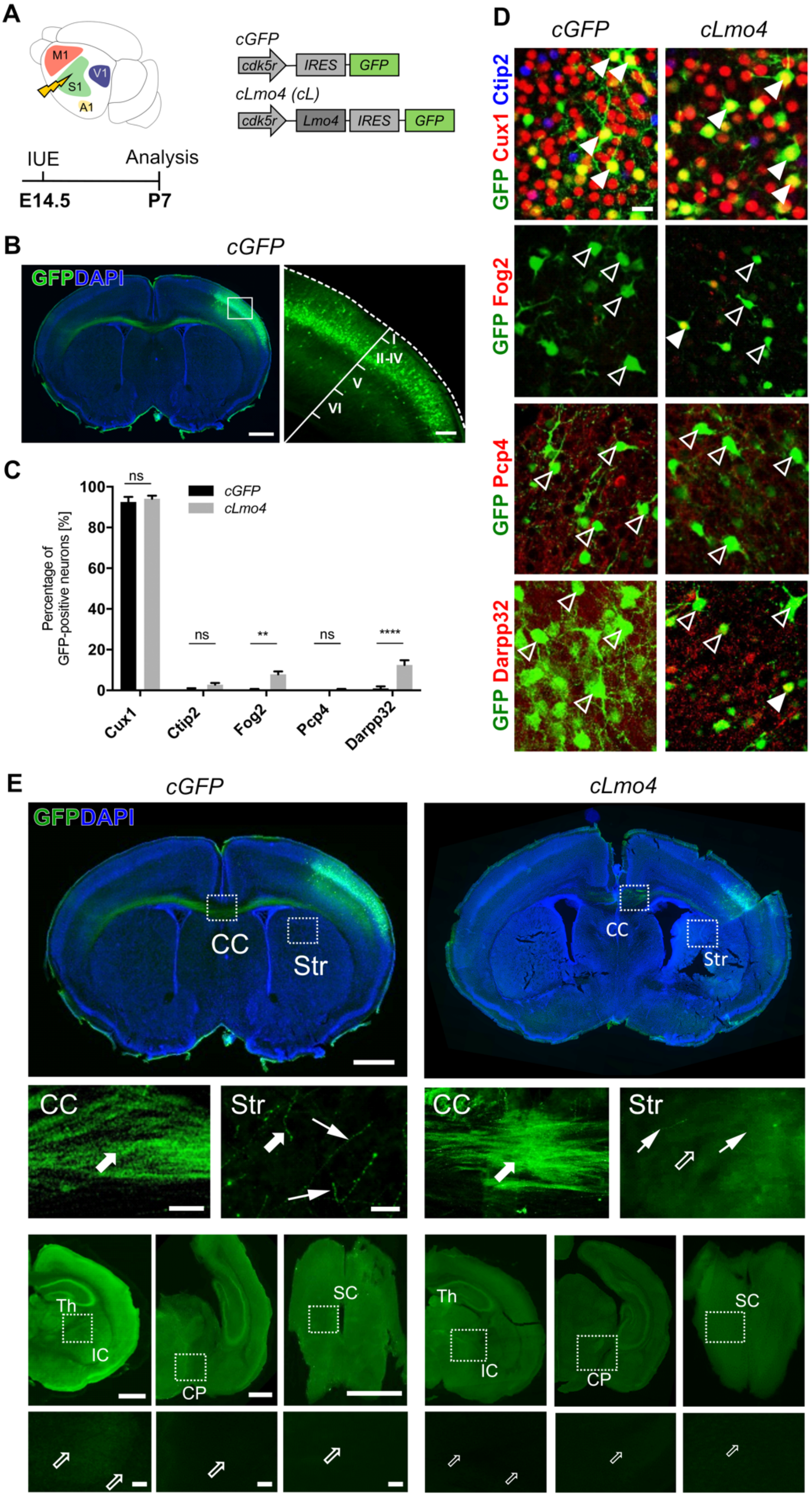


**S2 Fig: Overexpression of cLmo4 fails to lead to any lineage conversion and axonal rewiring. (A)** *Schematic representation of the experimental procedure and vectors. cGFP and cLmo4 (cL) plasmids were electroporated*

*into E14.5 somatosensory (S1) cortices. Brains were collected at P7.* ***(B)*** *Immunofluorescence (IF) of GFP on a coronal slice of an electroporated brain confirms the expected laminar localization (layers II-IV) of electroporated GFP+ cells. (**C**) Percentage of S1 electroporated UL neurons expressing upper versus deep-layer markers. (**D**) Representative images of Cux1, Ctip2, Fog2, Pcp4 and Darpp32 IF staining in electroporated brains. Full and empty arrowheads respectively indicate whether GFP+ cells co-express or not, respectively, the marker. (**E**) Tract tracing of upper layer GFP+ axons upon electroporation of cGFP and cLmo4 in P7 brains. No particular changes between cGFP- and cLmo4-electroporated brains have been detected. Scale bars: B= 1000µm (left, macro image) and 200µm (right, magnified image); D= 20µm; E= 1000µm (top row, macro images), 200µm (magnified images). Results are expressed as mean ± s.e.m. Two-way ANOVA with Tukey's post-hoc correction was used for statistical analysis, **p<0.01 ***p<0.0001. n = 3 brains for each plasmid. Abbreviations: CC, corpus callosum; CP, cerebral peduncle; IC, internal capsule; SC, spinal cord; Str, striatum; Th, thalamus. All individual data and statistics are listed in S1 Data. See also Table 1.*
