# Supplementary material for: Lmo4 synergizes with Fezf2 to promote direct in vivo reprogramming of upper layer cortical neurons and cortical glia towards deep-layer neuron identities
Source: PLoS Biol. 2023 Aug 8;21(8):e3002237. doi: 10.1371/journal.pbio.3002237 (PMC10409279; doi:10.1371/journal.pbio.3002237)
Supplement: S3 Fig — (A) Schematic representation of the experimental procedure and vectors used in this experiment. iFezf2 (iF) and iLmo4 (iL) were electroporated into E14.5 somatosensory (S1) cortices. Gene expression was induced at P3 by tamoxifen (TAM) subcutaneous injection. Brains were collected at P8. (B) Immunofluorescence (IF) of GFP, upper-layer marker Cux1 and deep-layer V marker Ctip2 on a coronal slice of an electroporated brain. White box indicates magnification image on the right side. (C) Validation of the correct expression of Fezf2 and Lmo4 proteins by IF of TAM-induced brains. Note that almost all GFP+ cells express Lmo4 and/or Fezf2. Below, confocal images of high-magnification panels showing 3D reconstructions of double staining. Side bars represent projections along the x–z axes (right) and the y–z axes (below). (D) No GFP expression is detected in NO-TAM-induced brains co-electroporated with mCherry (in red). Scale bars: B = 1,000 μm (left, macro image) and 200 μm (right, magnified image); C, D = 20 μm. (PDF) [file pbio.3002237.s003.pdf]

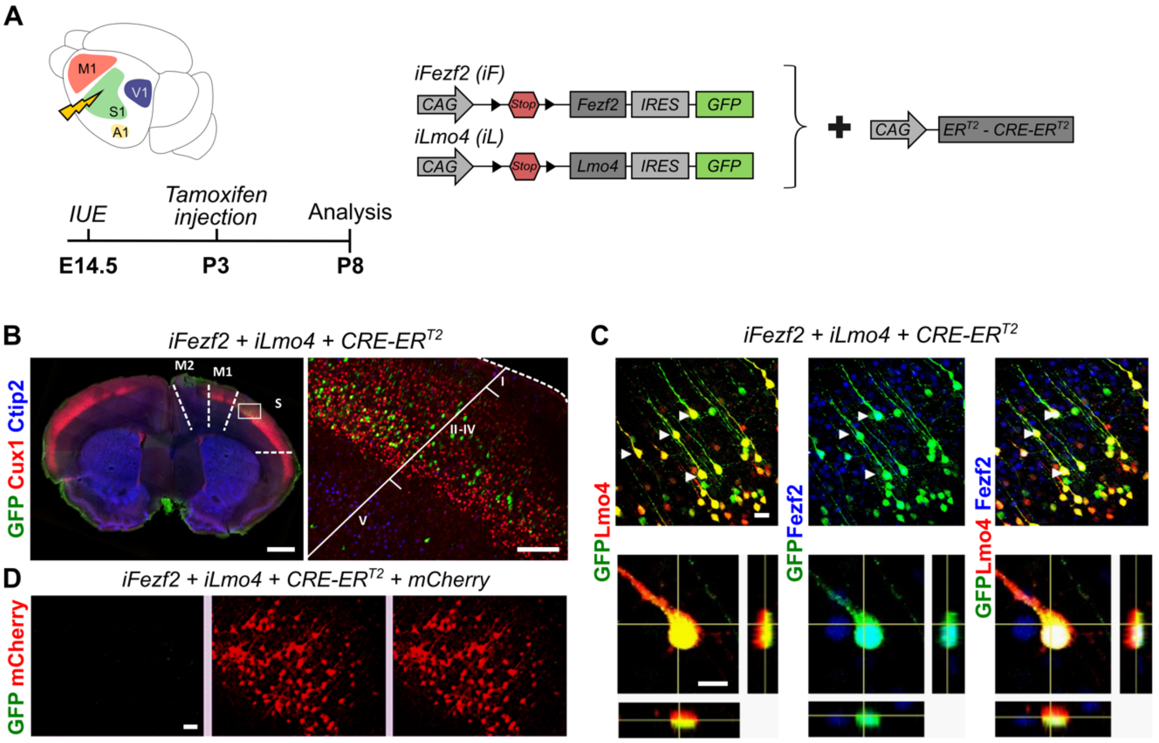

S3 Fig: iFezf2 and iLmo4 are properly expressed in induced electroporated brains. *(**A**) Schematic representation of the experimental procedure and vectors used in this experiment. iFezf2 (iF) and iLmo4 (iL) were electroporated into E14.5 somatosensory (S1) cortices. Gene expression was induced at P3 by tamoxifen (TAM) subcutaneous injection. Brains were collected at P8. (**B**) Immunofluorescence (IF) of GFP, upper layer marker Cux1, and deep layer V marker Ctip2 on a coronal slice of an electroporated brain. White box indicates the magnification image on the right side. **(C)** Validation of the correct expression of Fezf2 and Lmo4 proteins by IF of TAM-induced brains. Note that almost all GFP+ cells express Lmo4 and/or Fezf2. Below, confocal images of high-magnification panels showing three-dimensional reconstructions of double staining. Sidebars represent projections along the x–z axes (right) and the y–z axes (below). (**D**) No GFP expression is detected in NO-TAM-induced brains co-electroporated with mCherry (in red). Scale bars: B= 1000µm (left, macro image) and 200µm (right, magnified image); C, D= 20µm.*
