# Supplementary material for: Lmo4 synergizes with Fezf2 to promote direct in vivo reprogramming of upper layer cortical neurons and cortical glia towards deep-layer neuron identities
Source: PLoS Biol. 2023 Aug 8;21(8):e3002237. doi: 10.1371/journal.pbio.3002237 (PMC10409279; doi:10.1371/journal.pbio.3002237)
Supplement: S4 Fig — (A) Schematic representation of the experimental procedure and vectors. iGFP, iFezf2 (iF), or iFezf2 and iLmo4 (iF+iL) together with pCAG-CRE-ERT2 were electroporated into E14.5 somatosensory (S1) cortices. The smFP-Flag reporter plasmid was co-electroporated to facilitate axon tracing. Gene expression was induced at P7 by tamoxifen subcutaneous injection. Brains were collected at P14. (B) Tract tracing of upper-layer FLAG+ axons upon electroporation of iGFP, iF or, iF+iL vectors. Full and empty arrows indicate presence or absence of axons, respectively. FLAG+ axons were found crossing the corpus callosum (CC) in all conditions, but they reached the thalamus (Th), internal capsule (IC), and cerebral peduncle (CP) only in iF and iF+iL conditions; axons were clearly detected in the spinal cord (SC) of iF+iL brains. White boxes indicate regions magnified in the panels below or aside. Scale bars: B = 1,000 μm (macro images) and 20 μm (magnification images). n = 3 brains for each plasmid. Str, striatum. See also Table 1. (PDF) [file pbio.3002237.s004.pdf]

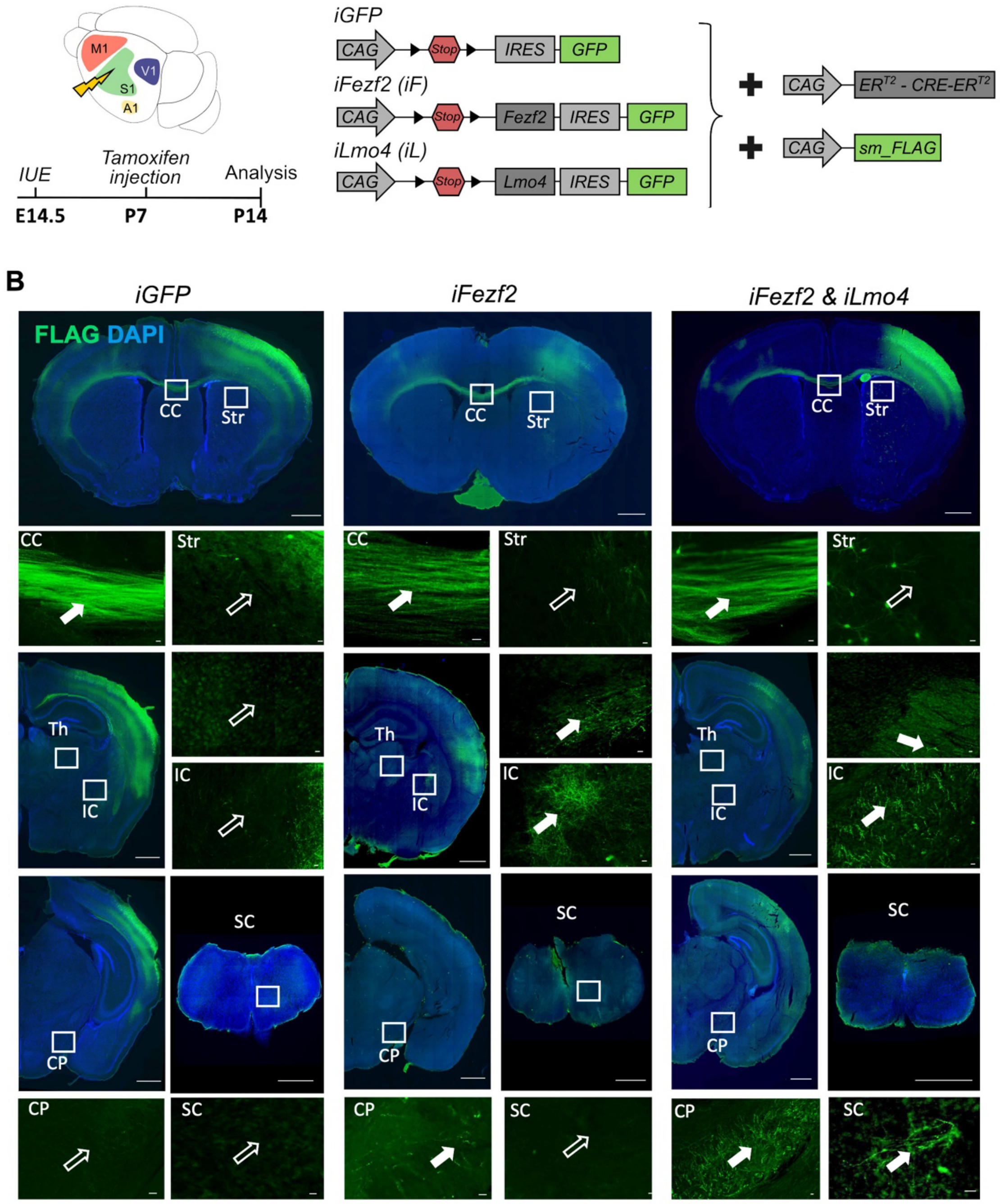

Fig S4. P7 induction of Fezf2 and Lmo4 expression can partially change upper-layer axonal projections toward subcerebral targets. *(**A**) Schematic representation of the experimental procedure and vectors. iGFP, iFezf2 (iF) or iFezf2 + iLmo4 (iF+iL) together with pCAG-CRE-ERT2 were electroporated into E14.5 somatosensory (S1) cortices. smFP-Flag reporter plasmid was co-electroporated to facilitate axon tracing. Gene expression was induced at P7 by tamoxifen subcutaneous injection. Brains were collected at P14. (**B**) Tract tracing of upper layer FLAG+ axons upon electroporation of iGFP, iF or iF+iL vectors. Full and empty arrows indicate the presence or absence of axons, respectively. FLAG+ axons were found crossing the corpus callosum (CC) in all conditions, but they reached the thalamus (Th), internal capsule (IC), and cerebral peduncle (CP) only in iF and iF+iL conditions; axons were clearly detected in the spinal cord (SC) of iF+iL brains. White boxes indicate regions magnified in the panels below or aside. Scale bars: B = 1000µm (macro images) and 20µm (magnification images). n = 3 brains for each plasmid. Abbreviations: Str, striatum. See also Table 1.*
