# Supplementary material for: Lmo4 synergizes with Fezf2 to promote direct in vivo reprogramming of upper layer cortical neurons and cortical glia towards deep-layer neuron identities
Source: PLoS Biol. 2023 Aug 8;21(8):e3002237. doi: 10.1371/journal.pbio.3002237 (PMC10409279; doi:10.1371/journal.pbio.3002237)
Supplement: S5 Fig — (A) Schematic representation of the experimental procedure and vectors. iGFP, iFezf2 (iF) or iFezf2, and iLmo4 (iF+iL) together with pCAG-CRE-ERT2 were electroporated into E14.5 somatosensory (S1) cortices. smFP-Flag reporter plasmid was co-electroporated to facilitate axon tracing. Gene expression was induced at P10 by tamoxifen subcutaneous injection. Brains were collected at P21. (B) Tract tracing of upper-layer FLAG+ axons upon electroporation of iGFP, iF, or iF+iL vectors. Full and empty arrows indicate the presence or absence of FLAG+ axons, respectively. Axons were found crossing the corpus callosum (CC) and reaching the striatum (Str) in all conditions, but their presence was detected in the internal capsule (IC) and cerebral peduncle (CP) only in iF- and iF+iL-electroporated brains. Although big bundles of axons can be observed in the CP of iF-electroporated brains, no obvious projections seem to reach the spinal cord (SC), differently from iF+iL-electroporated brains in which few dispersed axons ultimately reach the SC. White boxes indicate regions magnified in the panels below or aside. Scale bars: B = 1,000 μm (macro images) and 20 μm (magnification images). n = 3 brains for each plasmid. See also Table 1. (PDF) [file pbio.3002237.s005.pdf]

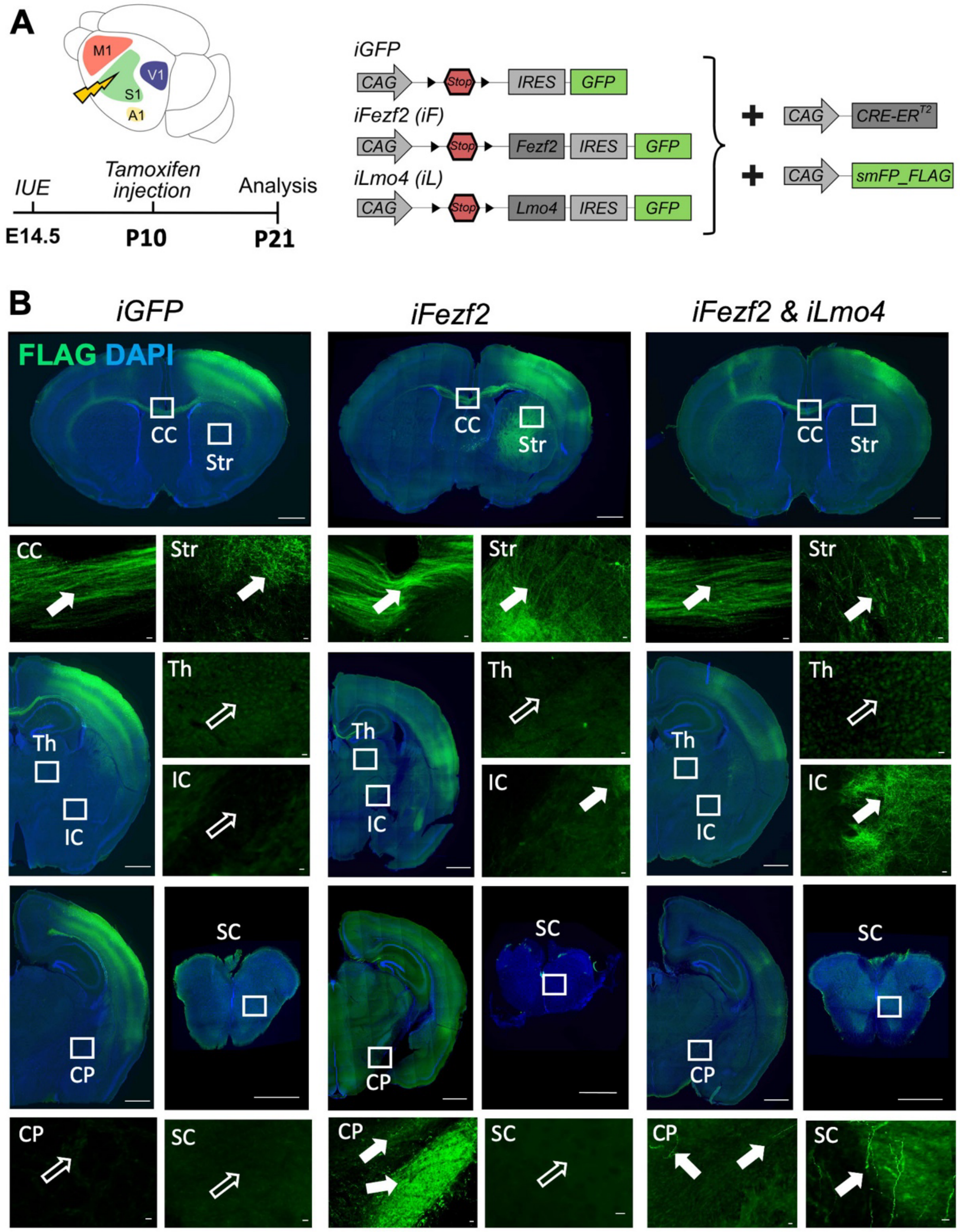

S5 Fig: P10 induction of double Fezf2 and Lmo4 expression can partially change upper-layer axonal projections toward subcerebral targets. *(**A**) Schematic representation of the experimental procedure and vectors. iGFP, iFezf2 (iF), or iFezf2 + iLmo4 (iF+iL) together with pCAG-CRE-ERT2 were electroporated into E14.5 somatosensory (S1) cortices. smFP-Flag reporter plasmid was co-electroporated to facilitate axon tracing. Gene expression was induced at P10 by tamoxifen subcutaneous injection. Brains were collected at P21. (**B**) Tract tracing of upper layer FLAG+ axons upon electroporation of iGFP, iF, or iF+iL vectors. Full and empty arrows indicate the presence or absence of FLAG+ axons, respectively. Axons were found crossing the corpus callosum (CC) and reaching the striatum (Str) in all conditions, but their presence was detected in the internal capsule (IC) and cerebral peduncle (CP) only in iF- and iF+iL-electroporated brains. Although big bundles of axons can be observed in the CP of iF-electroporated brains, no obvious projections seem to reach the spinal cord (SC), differently from iF+iL-electroporated brains in which few dispersed axons can ultimately reach the SC. White boxes indicate regions magnified in the panels below or aside. Scale bars: B = 1000µm (macro images) and 20µm (magnification images). n = 3 brains for each plasmid. See also Table 1.*
