# Supplementary material for: Lmo4 synergizes with Fezf2 to promote direct in vivo reprogramming of upper layer cortical neurons and cortical glia towards deep-layer neuron identities
Source: PLoS Biol. 2023 Aug 8;21(8):e3002237. doi: 10.1371/journal.pbio.3002237 (PMC10409279; doi:10.1371/journal.pbio.3002237)
Supplement: S6 Fig — (A) Schematic representation of the experimental procedure and vectors. iGFP, iFezf2 (iF) or iFezf2, and iLmo4 (iF+iL) together with pCAG-CRE-ERT2 were electroporated into E14.5 somatosensory (S1) cortices. smFP-Flag reporter plasmid was co-electroporated to facilitate axon tracing. Gene expression was induced at P21 by tamoxifen subcutaneous injection. Brains were collected at P35. (B) Tract tracing of upper-layer FLAG+ axons upon electroporation of iGFP, iF, or iF+iL vectors. Full and empty arrows indicate the presence or absence of FLAG+ axons, respectively. Axons were found crossing the corpus callosum (CC) and reaching the striatum (Str) and internal capsule (IC) in all conditions. However, labeled axons were only detected in the cerebral peduncle (CP) and spinal cord (SC) of iF- and iF+iL-electroporated brains. White boxes indicate regions magnified in the panels below or aside. Scale bars: B = 1,000 μm (macro images) and 20 μm (magnification images). n = 3 brains for each plasmid. See also Table 1. (PDF) [file pbio.3002237.s006.pdf]

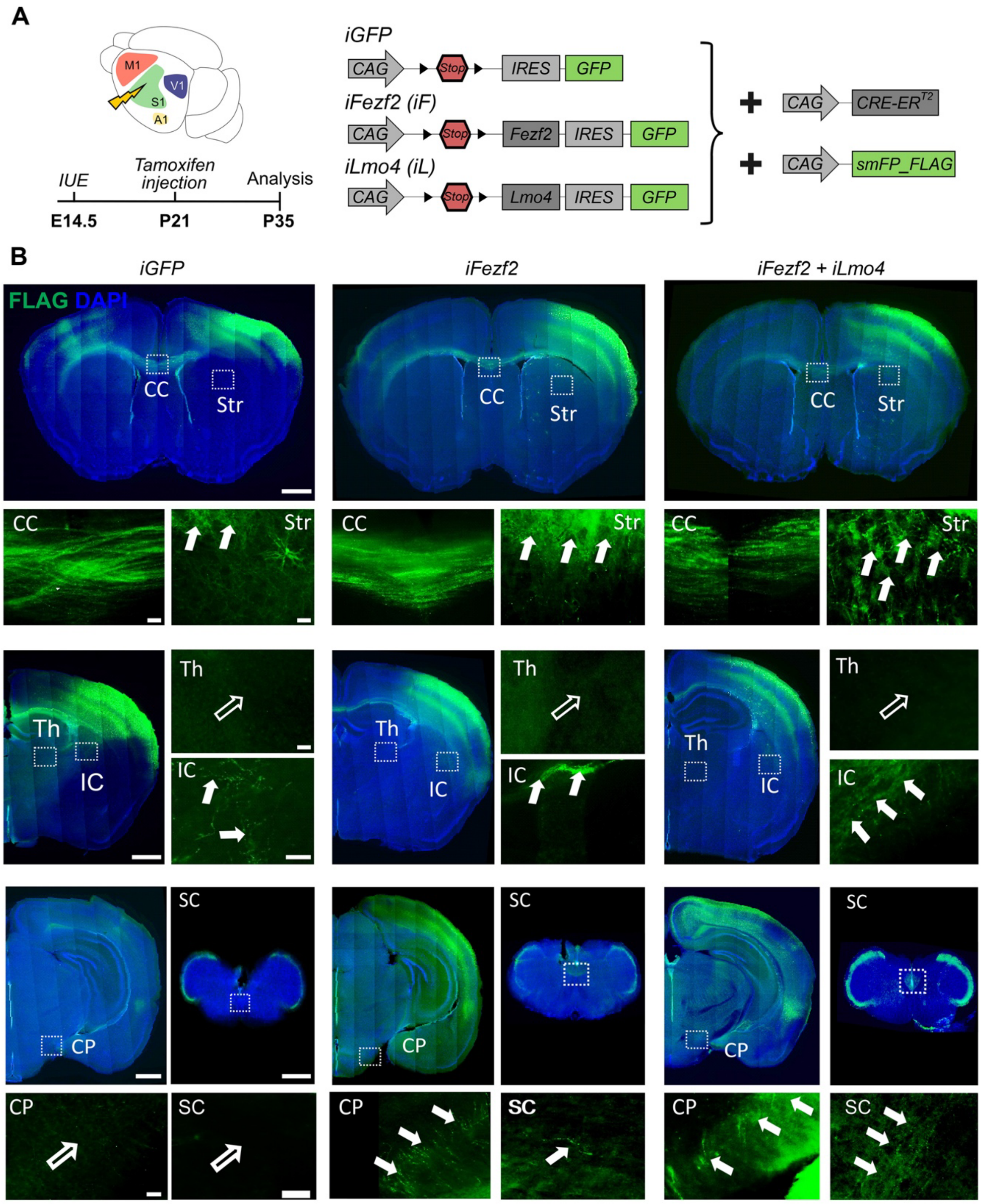

S6 Fig: P21 induction of single Fezf2 and double Fezf2 and Lmo4 expression can still partially change upper-layer axonal projections toward subcerebral targets. *(**A**) Schematic representation of the experimental procedure and vectors. iGFP, iFezf2 (iF) or iFezf2 and iLmo4 (iF+iL) together with pCAG-CRE-ERT2 were electroporated into E14.5 somatosensory (S1) cortices. smFP-Flag reporter plasmid was co-electroporated to facilitate axon tracing. Gene expression was induced at P21 by tamoxifen subcutaneous injection. Brains were collected at P35. (**B**) Tract tracing of upper layer FLAG+ axons upon electroporation of iGFP, iF, or iF+iL vectors. Full and empty arrows indicate the presence or absence of FLAG+ axons, respectively. Axons were found crossing the corpus callosum (CC) and reaching the striatum (Str) and internal capsule (IC) in all conditions. However, labeled axons were only detected in the cerebral peduncle (CP) and spinal cord (SC) of iF- and iF+iL -electroporated brains. White boxes indicate regions magnified in the panels below or aside. Scale bars: B = 1000µm (macro images) and 20µm (magnification images). n = 3 brains for each plasmid. See also Table 1.*
