# Supplementary material for: Lmo4 synergizes with Fezf2 to promote direct in vivo reprogramming of upper layer cortical neurons and cortical glia towards deep-layer neuron identities
Source: PLoS Biol. 2023 Aug 8;21(8):e3002237. doi: 10.1371/journal.pbio.3002237 (PMC10409279; doi:10.1371/journal.pbio.3002237)
Supplement: S7 Fig — (A) Analysis at 12 dpi of DCX-expression in NBF- and NBFL-cells with regards to their depth position (relative to the cortical surface) shows that reprogramming is induced at similar rates in all cortical layers. Apparent local accumulation of iNs in the first 200 μm below the cortical surface reflects higher numbers of transduced cells at this depth, but not a difference in the reprogramming efficiency (i.e., percentage of DCX-expressing cells among transduced cells). In the graphs, each dot represents a cell. n = 3 brains/retrovirus combination, 2,827 cells anaylzed. Images illustrate cell distribution in the cortex from the surface to deep-layers. All individual data are listed in S5 Data. (PDF) [file pbio.3002237.s007.pdf]

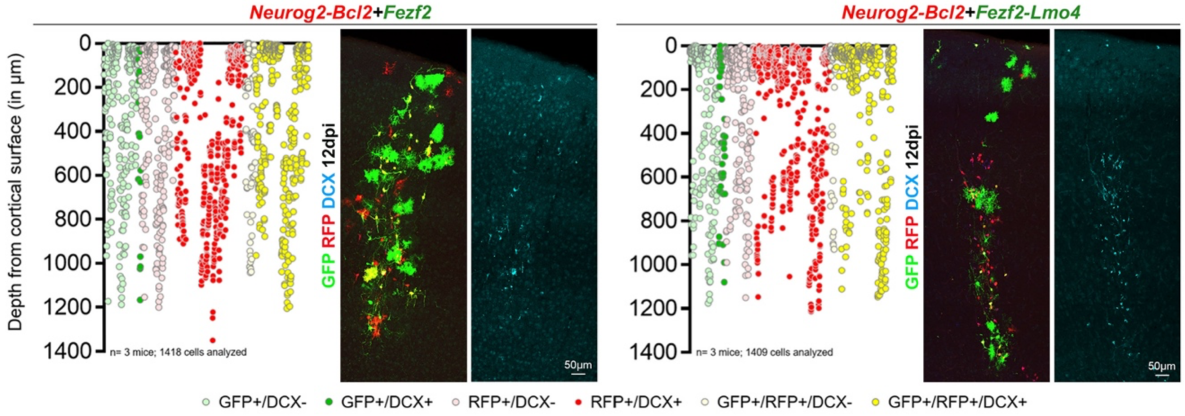

S7 Fig: NBF and NBFL induce glia-to-neuron reprogramming in all cortical layers. *(A) Analysis at 12dpi of DCX-expression in NBF- and NBFL-cells with regards to their depth position (relative to the cortical surface) shows that reprogramming is induced at similar rates in all cortical layers. Apparent local accumulation of iNs in the first 200µm below the cortical surface reflects higher numbers of transduced cells at this depth, but not a difference in the reprogramming efficiency (i.e., percentage of DCX-expressing cells among transduced cells). In the graphs, each dot represents a cell. n=3 brains/retrovirus combination, 2827 cells analyzed. Images illustrate cell distribution in the cortex from the surface to deep layers. All individual data are listed in S5 Data.*
