# Supplementary material for: Lmo4 synergizes with Fezf2 to promote direct in vivo reprogramming of upper layer cortical neurons and cortical glia towards deep-layer neuron identities
Source: PLoS Biol. 2023 Aug 8;21(8):e3002237. doi: 10.1371/journal.pbio.3002237 (PMC10409279; doi:10.1371/journal.pbio.3002237)
Supplement: S1 Table — (PDF) [file pbio.3002237.s014.pdf]

Antigen	Provider	Reference	Host	Use	Working Dilution
GFP	Abcam	ab13970	Chicken	IF – 1AB	1:500
GFP	AvesLab	GFP-1020	Chicken	IF – 1AB	1:1000
Flag	Novusbio	NBP1-0671255	Rat	IF – 1AB	1:200
Cux1	Merk-Millipore	ABE217	Rabbit	IF – 1AB	1:200
Ctip2	Abcam	ab18465	Rat	IF – 1AB	1:500
Fog2	Santa Cruz	sc-10755	Rabbit	IF – 1AB	1:200
PCP4	Thermo Fisher	PA5-52209	Rabbit	IF – 1AB	1:200
Darp32	Abcam	ab40801	Rabbit	IF – 1AB	1:200
Lmo4	Gift from J. Valsvader		Rat	IF – 1AB	1:500
Fezf2	Thermo Fisher	JP18997	Rabbit	IF – 1AB	1:200
RFP	Abcam	ab124754	Rabbit	IF – 1AB	1:200
RFP	Biomol	600401379S	Rabbit	IF – 1AB	1:500
Doublecortin (DCX)	Santa Cruz Biotechnology	sc-8066	goat	IF – 1AB	1:250
NeuN	Millipore	MAB377	mouse	IF – 1AB	1:500
Ck IgY - AF 488	Thermo Fisher	A11039	Goat	IF – 2AB	1:400
Rb IgG - AF 594	Thermo Fisher	A21428	Goat	IF – 2AB	1:400
Rat IgG - AF 647	Thermo Fisher	A21247	Goat	IF – 2AB	1:400
Rat IgG - AF 488	Thermo Fisher	A11006	Goat	IF – 2AB	1:400
Ck IgY – AF488	Jackson Immunoresearch	703-545-155	Donkey	IF – 2AB	1:200
Rb IgG – Cy3	Dianova	711-165-152	Donkey	IF – 2AB	1:500
Goat IgG – Cy5	Dianova	705-175-147	Donkey	IF – 2AB	1:500
Ms IgG – AF647	Invitrogen	A31571	Donkey	IF – 2AB	1:500
Rat IgG – FP647	Interchim	FP-SC6120	Donkey	IF – 2AB	1:500

S1 TABLE. Complete list of antibodies used in this study.
